# E-Freeze - a randomised controlled trial evaluating the clinical and cost effectiveness of a policy of freezing embryos followed by thawed frozen embryo transfer compared with a policy of fresh embryo transfer, in women undergoing in vitro fertilisation: a statistical analysis plan

**DOI:** 10.1186/s13063-020-04441-9

**Published:** 2020-06-30

**Authors:** Jennifer L. Bell, Pollyanna Hardy, Melanie Greenland, Edmund Juszczak, Christina Cole, Abha Maheshwari, Siladitya Bhattacharya, Louise Linsell

**Affiliations:** 1 University of Oxford, Oxford, UK; 2 University of Birmingham, Birmingham, UK; 3 NHS Grampian, Aberdeen, UK; 4 University of Aberdeen, Aberdeen, UK

**Keywords:** IVF, Fertility, Frozen thawed embryo transfer, Fresh embryo transfer, OHSS, Elective freezing, Assisted conception, Receptivity, Statistical analysis plan, Randomised controlled trial, Complier average causal effect

## Abstract

**Background:**

The E-Freeze trial is a multi-centre randomised controlled trial of fresh versus frozen embryo transfer for women undergoing in vitro fertilisation. This paper describes the statistical analysis plan for the E-Freeze trial.

**Methods and design:**

E-Freeze is a two-arm parallel-group, multi-centre, individually randomised controlled trial to determine if a policy of freezing embryos, followed by thawed frozen embryo transfer, results in a higher healthy baby rate when compared with the current policy of transferring fresh embryos. Couples undergoing their first, second or third cycle of in vitro fertilisation at fertility centres in the UK were randomised to either fresh or frozen embryo transfer. The primary outcome is a healthy baby, defined as a live singleton baby born at term with an appropriate weight for gestation. This paper describes the statistical analysis plan for the trial, including the analysis principles, definitions of outcomes, methods for primary analysis, pre-specified subgroup analysis and sensitivity analysis. This plan was finalised prior to completion of recruitment to the trial.

**Trial registration:**

ISRCTN registry: ISRCTN61225414. Registered on 29 December 2015.

## Introduction

This paper details the proposed presentation and analyses for the main paper(s) reporting results from the National Institute for Health Research (NIHR) Health Technology Assessment (HTA) programme-funded multicentre randomised controlled trial of fresh versus frozen embryo transfer for women undergoing in vitro fertilisation (E-Freeze).

E-Freeze is a multi-centre randomised controlled trial (RCT) to determine if a policy of freezing embryos, followed by thawed frozen embryo transfer, results in a higher healthy baby rate when compared with the current policy of transferring fresh embryos. Couples undergoing their first, second or third cycle of in vitro fertilisation at fertility centres in the UK were randomised to either fresh or frozen embryo transfer. This paper describes the statistical analysis plan for the main trial, including the analysis principles, definitions of outcomes, methods for primary analysis, pre-specified subgroup analysis and sensitivity analysis.

This statistical analysis plan conforms to the published guidelines on the content for statistical analysis plans in clinical trials [1], and was finalised prior to completion of recruitment to the trial. Any deviations from this plan will be described and justified in the final report of the trial.

## Background Information

### Rationale

In vitro fertilisation (IVF) involves several steps. Initially, hormones are used to stimulate a woman’s ovaries to produce eggs, which are harvested surgically. Next, embryos are created in the laboratory by mixing eggs with sperm produced by her partner. In conventional IVF, these are grown in culture for a few days before being replaced within the uterus by a process known as fresh embryo transfer. Spare embryos are usually frozen with a view to transfer at a later point in time - especially if the initial fresh transfer does not result in a pregnancy. Despite improvements in technology, IVF success rates remain low with an overall live birth rate of 25% per treatment. Additionally, there are concerns about health outcomes for mothers and babies conceived through IVF, particularly after fresh embryo transfer, including maternal ovarian hyperstimulation syndrome (OHSS) and perinatal morbidity.

It is believed that high levels of ovarian hormones during ovarian stimulation could create a relatively hostile environment for embryo implantation whilst increasing the risk of OHSS. It has been suggested that electively freezing embryos with the intention of thawing and replacing them within the uterus at a later stage (thawed frozen embryo transfer) instead of fresh embryo transfer, may lead to improved pregnancy rates and fewer complications. However, at the time of developing this study, the evidence in support of an elective frozen embryo transfer policy was inadequate to justify such a radical change in practice. In addition, there are risks identified both for mother and babies in pregnancies as a result of frozen embryo transfer.

The E-Freeze trial aims to determine if a policy of freezing embryos, followed by thawed frozen embryo transfer, results in a higher healthy baby rate when compared with the current policy of transferring fresh embryos. The target sample size of the trial was 1086 couples undergoing their first, second or third cycle of IVF treatment at fertility centres in the UK [2].

### Objectives of the trial

The primary objective of the trial is to determine if a policy of freezing embryos, followed by thawed frozen embryo transfer, results in a higher healthy baby rate when compared with the current policy of transferring fresh embryos. However, so as not to preclude the possibility that the latter results in a higher healthy baby rate than the former, our primary objective is evaluated by presenting two-sided confidence intervals.

The secondary objectives of the trial are to compare the number of complications associated with IVF/intracytoplasmic sperm injection (ICSI) treatment and pregnancy, and the cost-effectiveness from a health service and broader societal perspective.

## Study methods

### Trial design

This is a multi-centre, two-arm, parallel-group individually randomised controlled trial.

### Eligibility

Inclusion criteria:

- The female partner is ≥ 18 and < 42 years of age at the start of treatment (i.e. start of ovarian stimulation)
- Couples who are undergoing their first, second or third cycle of IVF or ICSI treatment
- Both partners are resident in the UK
- Both partners have provided written informed consent
- At least three good-quality embryos on day 3 after fertilisation

Exclusion criteria:

- Donor gametes are used
- Pre-implantation genetic testing is planned
- Elective freezing of all embryos is planned for medical reasons (e.g. severe risk of OHSS/fertility preservation)
- Couples previously randomised to E-Freeze

### Interventions

Standard care arm: women will undergo fresh embryo transfer at the cleavage (day 3 of embryo development) or blastocyst stage (day 5 of embryo development) according to local fertility centre protocols. Intervention arm: all good quality embryos will be frozen according to local fertility centre protocols.

Women will be contacted by their embryologist/research nurse (or delegate) after randomisation and arrangements will be made for frozen embryo transfer (typically after 4–6 weeks and always within 3 months of the egg retrieval process). The couples will attend for a clinic visit and additional monitoring visits before thawed frozen embryo transfer is performed.

### Definition of primary and secondary outcomes

#### Primary outcome

The primary outcome is a healthy baby, defined as a live singleton baby born at term (between 37 and 42 completed weeks of gestation) with an appropriate weight for gestation (weight between 10th and 90th centile for that gestation based on standardised charts).

#### Secondary outcomes

The secondary outcomes are grouped as follows: maternal safety, complications of pregnancy and delivery, clinical effectiveness, effectiveness of the process of freezing embryos and health economic outcome measures.

#### Maternal safety outcome

The maternal safety outcome is OHSS - defined and classified as per the Royal College of Obstetricians and Gynaecologists (RCOG) green-top guidelines [3].

#### Complications of pregnancy and delivery outcomes

Outcomes measuring complications of pregnancy and delivery are:

- Vanishing twin or triplet (defined as more fetal heartbeats than babies born, more gestational sacs than babies born or more gestational sacs than fetal heartbeats)
- Miscarriage rate (defined as pregnancy loss prior to age of viability i.e. 24 weeks of gestation)
- Ectopic pregnancy
- Termination
- Gestational diabetes mellitus (GDM)
- Multiple pregnancy (defined as more than one fetal heartbeat or more than one gestational sac)
- Multiple births (including live and still births)
- Hypertensive disorders of pregnancy (chronic hypertension, pregnancy-induced hypertension, pre-eclampsia and eclampsia)
- Most severe hypertensive disorder (from least to worst: chronic hypertension, pregnancy-induced hypertension, pre-eclampsia and eclampsia)
- Antepartum haemorrhage (any bleeding *per vaginum* after 28 weeks of pregnancy including placenta praevia and placental abruption)
- Onset of labour (spontaneous, induced or planned caesarean section)
- Mode of delivery for each baby (normal vaginal delivery, instrumental vaginal delivery or caesarean section)
- Preterm delivery (defined as delivery at < 37 completed weeks)
- Very preterm delivery (defined as delivery at < 32 completed weeks)
- Low birth weight (defined as weight < 2500 g at birth)
- Very low birth weight (defined as weight < 1500 g at birth)
- High birth weight (defined as weight > 4000 g at birth)
- Large for gestational age (defined as birth weight > 90th centile for gestational age at delivery, based on standardised charts)
- Small for gestational age (defined as birth weight < 10th centile for gestational age at delivery, based on standardised charts)
- Congenital anomaly/birth defect (all congenital anomalies/birth defects identified will be included)
- Perinatal mortality (stillbirth or late and early neonatal deaths, up to 28 days after birth)

#### Clinical effectiveness outcomes

Outcomes measuring clinical effectiveness are:

- Live birth rate (this is a live birth episode i.e. multiple births will count as one)
- Singleton live birth rate
- Singleton live birth rate at term
- Singleton baby with appropriate weight for gestation
- Pregnancy rate (defined as positive pregnancy test at 2 weeks ± 3 days after embryo transfer)
- Clinical pregnancy rate (defined as the presence of at least one fetal heartbeat at ultrasound between 6 and 8 weeks’ gestation; ectopic pregnancy counts as a clinical pregnancy; multiple gestational sacs count as one clinical pregnancy)

#### Effectiveness of the process of freezing embryos outcomes

Outcomes measuring the effectiveness of the process of freezing embryos are:

- Total number of embryos frozen, thawed and transferred for all randomised couples
- Proportion of thawed embryos that were then transferred for all randomised couples
- Failure of all embryos to survive after thawing leading to no embryo transfer

#### Health economic outcomes

The health economic outcomes are:

- Cost to the health service of treatment, pregnancy and delivery care
- Modelled long-term costs of health and social care, and broader societal costs

#### Other secondary outcomes

The final secondary outcome is the evaluation of emotional state (for both partners separately).

### Hypothesis framework

This is a superiority trial and all comparisons will be analysed and presented on this basis.

### Sample size and power

The proposed primary outcome for this trial is novel and is not currently reported by IVF clinics or national regulatory bodies. This means that a number of assumptions have been made in order to determine the expected event rate in the control arm (receiving current standard treatment), which may in turn result in a degree of imprecision in the estimate.

The most recent data from the Human Fertilisation and Embryology Authority (HFEA) [4], which collects data on all IVF cycles from all clinics in the UK, show that 25% of all women undergoing one episode of IVF treatment involving a fresh embryo transfer have a live birth, and 20% have singleton live births. These figures are for women of all age groups, not necessarily for women fulfilling the inclusion criteria for this trial in terms of the number of good-quality embryos in their IVF cycle. No data are available on the healthy baby rate (live singletons born between 37 and 42 weeks, with appropriate weight for gestation), the primary outcome for this study. For our trial population we anticipate that the control arm event rate is likely to be < 25%, possibly as low as 17%.

To provide relevant information on the event rate expected in the control arm, we surveyed ten IVF centres that expressed an interest in the study, collecting data on the number of live births in women under the age of 42 years undergoing their first IVF treatment in 2012. The average live birth episode rate from this survey was 31% with a 95% confidence interval of 25% to 37%. Accurate data on the healthy baby rate in those with at least three good-quality embryos were not available. Although the live birth rate is expected to be higher in women with at least three good-quality embryos (likely to have a better prognosis), we anticipate that the healthy baby rate in our trial population will be towards the lower end of the confidence interval, around 25%, taking into account the higher risk of preterm delivery and babies who are small for gestational age, following IVF [5].

The following assumptions have been made for the sample size calculation: we have assumed a healthy baby rate of between 17% and 25% in women eligible for the trial (age under 42 years with three good-quality embryos) undergoing standard care (fresh embryo transfer). Taking into account the extra time, effort and potential expense involved in freezing embryos and the delay in embryo transfer of up to 3 months, a panel of clinicians across the UK agreed that the strategy of freezing embryos would be considered effective if the percentage of women having a healthy baby is increased by at least 8% in absolute terms. With 90% power and using a two-sided 5% level of statistical significance, we will need to randomise a total of 1086 couples (543 in each group) in order to be able to detect an absolute difference of 8% from 17% to 25% and 9% from 25% to 34% in the healthy baby rate, between fresh embryo transfer and transfer of thawed frozen embryos. The difference detectable differs slightly depending on the event rate in the standard care group, which will be reviewed periodically by the Data Monitoring Committee (DMC).

It is a regulatory requirement for clinics in the UK to report live birth outcomes (including number, weight and gestation) after all embryo transfers i.e. there will be no loss to follow up. Therefore, we have not taken into account loss to follow up for these sample size calculations. Couples who withdraw consent to use data already collected will be defined as post-randomisation exclusions, and their outcome data will not be included - however, based on extensive experience, withdrawal rates are also expected to be very low, and so these have not been taken into account in the sample size calculations.

It is anticipated that a proportion of those consented may not reach randomisation (not having three good-quality day-3 embryos or requiring all embryos to be frozen for medical reasons), therefore a larger number will need to be consented. As there are no valid data to support the exact proportion, this will be monitored by the DMC.

### Intervention allocation

Randomisation will be performed after the creation of embryos, 3 days post egg collection. This will minimise the time interval from randomisation to intervention, as embryos are either transferred at the cleavage or blastocyst stage. Once all eligibility criteria are established (including ensuring that three or more good-quality embryos are available), women will be randomised (allocation ratio 1:1) to a strategy of either:

1. Fresh embryo transfer, or
2. Elective freezing of embryos followed by thawing and replacement at a later date (typically 4–6 weeks later and always within 3 months of egg collection)

Randomisation will be undertaken by the research nurse or a delegated member of the research team using a secure web-based centralised system (with 24-h/7 days/week telephone backup 365 days/year) hosted by the National Perinatal Epidemiology Unit Clinical Trials Unit (NPEU CTU), University of Oxford (ensuring allocation concealment). Participants will be randomised using a minimisation algorithm [6], with a random element to balance across the following factors: fertility clinic; woman’s age at start of treatment (< 35 years, 35 to < 40 years, ≥ 40 years); primary/secondary infertility; self-reported duration of infertility (< 12 months, 12 to < 24 months, 24 to < 36 months, 36 to < 48 months, 48 to < 60 months, ≥ 60 months); method of insemination (IVF, ICSI or a combination of both) and number of previous egg collections (0, 1 or 2 cycles). The implementation of the randomisation procedure will be monitored by the Senior Trials Programmer throughout the trial and reports will be provided to the DMC.

### Data collection schedule

Data for both clinical and economic outcomes will be collected using bespoke electronic case report forms (eCRFs) and entered directly into the study OpenClinica electronic database by the centre research staff.

**Fig. 1:**
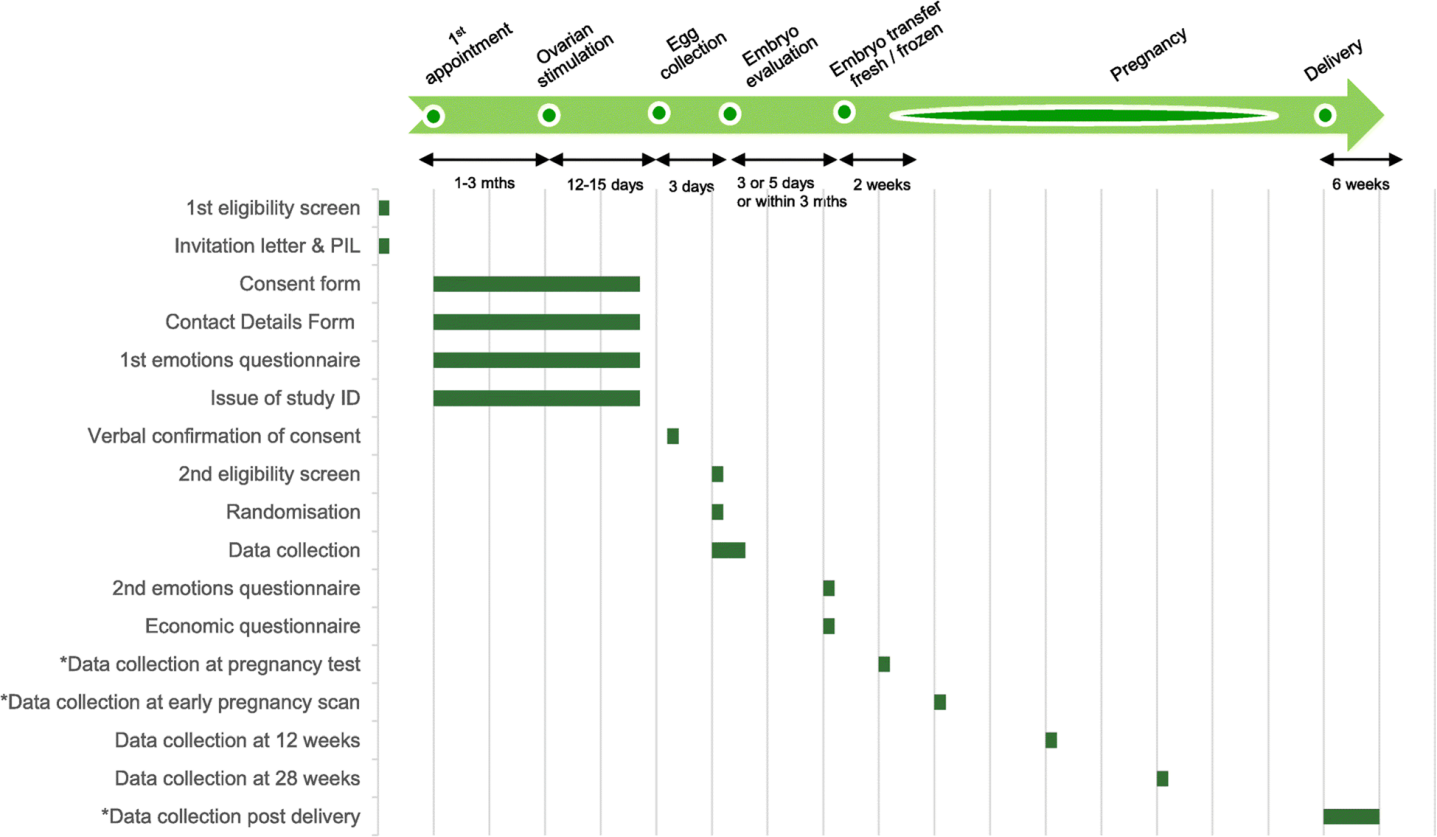
Data collection schedule

After consent and at embryo transfer, the couples will each complete a short paper-based questionnaire asking them how they are feeling. A short questionnaire will be provided for each partner to record details of time and travel expenses accrued during their treatment as part of the economic evaluation. This is to be completed at the time of embryo transfer. See Fig. 1 for the data collection schedule.

### Interim analyses and stopping rules

An independent DMC has been established, whose remit will be to safeguard the interests of trial participants, potential participants, their families, their carers, investigators and the Sponsor; to assess the safety and efficacy of the intervention during the trial, and monitor the trial overall conduct, and protect its validity and credibility. The terms of reference for the DMC were agreed at their first meeting and documented in a DMC Charter.

Interim analyses will be supplied, in strict confidence, to the DMC, as frequently as the DMC Chair requests. The DMC will aim to meet in person at least annually or more often as appropriate, and meetings should be timed so that reports from the DMC can be fed into the Trial Steering Committee (TSC).

In the light of interim data and other evidence from relevant studies, the DMC will inform the TSC if, in its view, there is proof beyond reasonable doubt that the data indicate that the trial should be terminated. A decision to inform the TSC of such a finding will in part be based on statistical considerations. Appropriate proof beyond reasonable doubt cannot be specified precisely. A difference of at least three standard errors in the interim analysis of a major endpoint may be needed to justify halting or modifying the study prematurely. Unless modification or cessation of the trial is recommended by the DMC, the TSC, investigators, collaborators and administrative staff (except those who supply the confidential information) will remain ignorant of the results of the interim analysis. Collaborators and all others associated with the study may write to the DMC to draw attention to any concern they may have about the possibility of harm arising from the treatment under study. The Trial Statistician will provide the DMC with interim reports unblinded to allocation.

### Trial reporting

The trial will be reported according to the principles of the Consolidated standards of reporting trials (CONSORT) statement [7]. The final analysis will be conducted after follow up has been completed for the last woman to deliver.

## Protocol non-compliance

Protocol non-compliance is defined as a failure to adhere to the protocol such as the wrong intervention being administered, incorrect data being collected and documented, errors in applying inclusion/exclusion criteria or missed follow-up visits due to error. All instances of protocol non-compliance will be listed in the final report. Non-compliance is defined below.

### Major

Major protocol non-compliance will have a direct bearing on the primary outcome and in this study is pre-defined to be data considered to be fraudulent.

### Minor

The following will be defined as minor protocol non-compliance:

- Participants randomised in error. These include couples:
  - Where the female partner is not ≥ 18 and < 42 years of age at the start of treatment (i.e. start of ovarian stimulation);
  - Who are not undergoing their first, second or third cycle of IVF or ICSI treatment
  - who are not both resident in the UK;
  - For whom written informed consent is not provided from both partners;
  - Who have fewer than three good-quality embryos and/or the quality of embryos is assessed before or after day-3 following fertilization;
  - For whom donor gametes are used;
  - For whom pre-implantation genetic testing is planned;
  - For whom elective freezing of all embryos is planned for medical reasons (e.g. severe risk of OHSS/fertility preservation);
  - Who have previously been randomised to E-Freeze.

- Participants who do not receive their intervention as allocated. These include couples:
  - Randomised to the freezing of embryos followed by thawing and replacement at a later date (within 3 months of the date of egg retrieval) but who do not receive frozen embryo transfer or receive it after 3 months of the date of egg retrieval (defined as 3 calendar months);
  - Who switch from their randomised trial arm to the other arm;
  - Who do not receive any embryo transfer within 3 calendar months of the date of egg retrieval.

- Other minor protocol non-compliance. This includes couples:
  - Randomised before or after day-3 after egg collection (where day-0 is defined as the day of egg collection);
  - For whom a pregnancy test was carried out outside 2 weeks ± 3 days after embryo transfer;
  - For whom an ultrasound was performed before 6 weeks or after 8 weeks gestation;
  - Contacted by telephone outside 12 weeks gestation ± 14 days;
  - Contacted by telephone outside 28 weeks gestation ± 14 days.

## Adherence to the intervention

Data on adherence to the allocation will be collected. If non-adherence is reported, the reason will be recorded. Sensitivity analysis will be conducted to compare the outcomes in compliers and non-compliers using a complier-average causal effect (CACE) analysis (see “Sensitivity analysis” for details).

The date of egg collection and embryo transfer will be collected to derive the time to embryo transfer: the maximum length of time allowed for this as specified by the protocol is 3 months for frozen embryo transfers; we will report any non-adherence to this time limit.

## Analysis populations

### Post-randomisation exclusions

Exclusions to the analysis post randomisation are defined as any of the following:

- Couples for whom written consent forms from both partners were not received
- Couples for whom consent to use their data was withdrawn by one or both of the partners
- Couples for whom an entire record of fraudulent data was detected (should fraudulent data be detected, consideration will be given to excluding all data for the site where such data were found)

The numbers of post-randomisation exclusions will be reported by randomised treatment group in a figure presenting the flow of participants, and the reasons will be summarised.

## Population definitions

### Intention-to-treat population

The intention-to-treat (ITT) population will be all couples randomised, except for post-randomisation exclusions.

### Delivery population

This population will be all couples randomised who delivered at least one baby, except for post-randomisation exclusions. Baseline characteristics (demographic and clinical characteristics at trial entry) and post-randomisation clinical characteristics of the embryo and the endometrium will be presented for these couples.

### Interim analysis population

Baseline characteristics will be presented for all couples with available data at the time of the database snapshot, except for known post-randomisation exclusions. Secondary outcomes will be presented for all couples with available outcome data and women who have delivered at the time of the database snapshot, except for known post-randomisation exclusions.

### Safety population

The safety population will comprise all couples randomised, except for post-randomisation exclusions. Since the post-randomisation exclusions are couples for whom no written consent has been obtained or who have withdrawn permission for us to use their data, the safety population must exclude these couples.

## Descriptive analyses

### Loss to follow up

The number and percentage of missing outcome data among couples will be reported for the two trial arms in the results tables. All maternal and infant deaths will be reported separately.

As it is a regulatory requirement for clinics in the UK to report live birth outcomes after all embryo transfers, there will be no loss to follow up for the primary outcome.

### Representativeness of trial population and participant throughput

The flow of participants through each stage of the trial will be summarised by randomised group using a figure presenting the flow of participants, in accordance with the CONSORT 2010 statement [7]. This will describe the numbers of couples:

- Consented
- Not randomised (with reasons)
- Randomised
- Allocated to frozen embryo transfer
  - Received frozen embryo transfer
  - Did not receive frozen embryo transfer
    - ◦ Received fresh embryo transfer (with reasons)
    - ◦ No embryos transferred (with reasons)
- Allocated to fresh embryo transfer
  - Received fresh embryo transfer
  - Did not received fresh embryo transfer
    - ◦ Received frozen embryo transfer (with reasons)
    - ◦ No embryos transferred (with reasons)
- Randomised in error
- Withdrawn (consent to use data)
- Included in the ITT population
  - With positive pregnancy test
  - With early pregnancy scan
  - Delivered (number of women and number of babies)
- Post-randomisation exclusions (with reasons)

### Baseline comparability of randomised groups

Demographic and clinical characteristics at trial entry will be described for all couples in the ITT population by randomised group for (1) all couples in the ITT population and (2) for couples for whom at least one baby was delivered. The following characteristics will be described:

- Fertility clinic
- Woman’s age at ovarian stimulation (years)
- Woman’s ethnicity
- Woman’s smoking status
- Woman’s body mass index (BMI) (weight in kilograms/height in metres squared)
- Type of infertility
- Woman’s previous pregnancies
- Woman’s previous live births
- Main cause of infertility
- Duration of infertility (months)
- Endoscratch performed
- Stimulation regimen used
- Total stimulation dose of follicle-stimulating hormone (FSH) (international units)
- Adjuvants used
- Blood test performed on day of trigger injection
- Trigger injection used
- Total number of eggs collected
- Method of insemination
- Number of eggs fertilised normally (two pro nucleus)
- Time lapse used
- Good-quality embryos created on day 3
- Number of previous egg collections
- Number of previous embryo transfers

Clinical characteristics of the embryo and the endometrium, which are collected post-randomisation, will also be described for the ITT population by randomised group for (1) all couples in the ITT population and (2) for couples for whom at least one baby was delivered:

- Method of embryo freezing
- Number of embryos frozen
- Time from egg collection to embryo freezing
- Number of embryos thawed
- Stage of embryo at transfer
- Number of embryos transferred
- Number of remaining frozen embryos after transfer
- Method of endometrial preparation for transfer
- Endometrial appearance
- Endometrial thickness (millimetres)

The number and percentage will be presented for binary and categorical variables. The mean and standard deviation or the median and the interquartile range will be presented for continuous variables, or the range if appropriate. There will be no tests of statistical significance performed nor confidence intervals calculated for differences between randomised groups on any baseline variable.

### Description of compliance to intervention

Compliance to the allocated intervention will be presented for the ITT population. Compliance will be summarised by randomised group in a figure presenting the flow of participants as follows.

The number of women allocated to frozen embryo transfer who received fresh embryo transfer or no embryos will be reported, along with the reason if provided. This will include the number with embryos failing to survive after thawing.

The number of women allocated to fresh embryo transfer who received frozen embryo transfer within 3 calendar months from the date of egg collection, or no embryos will be reported, along with the reason if provided.

In addition, the number of women receiving frozen embryo transfer (whether allocated or not) within 3 months of egg collection will be presented in a table, along with the mean and standard deviation or median and interquartile range of the time from egg collection to embryo transfer for those receiving frozen embryo transfer. The number of women receiving fresh embryo transfer (whether allocated or not) will also be presented.

## Comparative analysis

Couples and babies will be analysed according to their allocation regardless of the intervention they actually received. The fresh embryo transfer group will be used as the reference group in all analyses.

Since we are following up women from prior to pregnancy until after pregnancy, this raises the question of the most appropriate denominator for outcomes that can only occur in particular pregnancy states. There will be some outcomes that cannot be reported for some women (e.g. miscarriage for women who do not become pregnant, birthweight of babies for women who had a miscarriage). In order to perform the analyses for all outcomes on the ITT analysis population, to maintain the randomised groups and avoid bias, these will not be considered as missing data and for these outcomes all women will be included in the denominator. This means that regardless of whether a pregnancy or live birth occurs, the woman will be included in the denominator once for all outcomes. Where this is a perinatal outcome, but there is no baby in whom the outcome can occur, the woman will still be included once in the denominator.

Outcomes will be summarised with counts and percentages for categorical variables, means and standard deviations for normally distributed continuous variables, or median and interquartile range for other non-normally distributed continuous variables.

Adjusted risk ratios and confidence intervals will be calculated using binomial regression with a log link, and if a model fails to converge, a Poisson regression model with a robust variance estimator will be used [8]. Analyses will be adjusted for all minimisation factors where possible (i.e. fertility clinic, woman’s age at the time of start of treatment (i.e. ovarian stimulation), primary/secondary infertility, self-reported duration of infertility, method of insemination and number of previous egg collections). Fertility clinic will be fitted with a randomly varying intercept in the model, and all other factors as fixed effects. Both crude and adjusted risk ratios will be presented, but the primary inference will be based on the adjusted estimates.

For neonatal secondary outcomes, the unit of analysis in the ITT analysis will be the mother. In cases of multiple pregnancy where the infants’ outcomes differ, the worst outcome will be reported.

Analysis of secondary outcomes will be clearly delineated from the primary outcomes in any statistical reports produced.

The emotions questionnaires at randomisation and post-embryo transfer capture responses from the State-Trait Anxiety Inventory (STAI) [9]. Analysis of covariance (ANCOVA) will be used to investigate if there is a post-embryo transfer difference in the means of the two treatment groups, adjusting for baseline. To avoid bias, maximise the power of the study and adhere to the ITT principle, the missing-indicator method [10] will be used to replace missing baseline scores. This method replaces all missing baseline observations with the same value and an extra indicator variable is added to the model to indicate whether the value for that variable is missing. Models will be fitted separately for both partners.

The following secondary outcomes will be summarised only and no significance testing will be conducted:

- Chronic hypertension, pregnancy induced hypertension, pre-eclampsia and eclampsia
- Most severe hypertensive disorder (from least to worst: chronic hypertension, pregnancy induced hypertension, pre-eclampsia and eclampsia)

### Detailed definition of outcomes

See Additional file 1: Appendix A for detailed definitions of the outcomes.

### Primary analysis

The primary analysis for the primary outcome and all secondary outcomes will be conducted on the ITT population.

### Secondary analyses

There are a number of other clinically relevant populations of importance in the interpretation of results. In order to assess the treatment effect for the clinically relevant populations (e.g. effect on live birth rate for women who become pregnant), secondary analyses of specific outcomes will be performed with respect to clinically relevant denominators. These are as follows:

Per total number of women with a positive pregnancy test at 2 weeks ± 3 days after embryo transfer:

- Miscarriage rate (defined as pregnancy loss prior to age of viability i.e. 24 weeks of gestation)
- Multiple pregnancy (defined as more than one fetal heartbeat or more than one gestational sac)

Per total number of pregnant women with an ongoing pregnancy resulting in delivery:

- Gestational diabetes mellitus
- Multiple births (including live and still births)
- Hypertensive disorders of pregnancy (chronic hypertension, pregnancy induced hypertension, pre-eclampsia and eclampsia)
- Most severe hypertensive disorders (from least to worst: chronic hypertension, pregnancy induced hypertension, pre-eclampsia and eclampsia)
- Antepartum haemorrhage (any bleeding *per vaginum* after 28 weeks of pregnancy including placenta praevia and placental abruption)
- Preterm delivery (defined as delivery at < 37 weeks completed)
- Very preterm delivery (defined as delivery at < 32 weeks completed)
- Onset of labour (spontaneous, induced or planned caesarean section)
- Mode of delivery for each baby (normal vaginal delivery, instrumental vaginal delivery or caesarean section)

Per total number of babies born:

- Low birth weight (defined as weight < 2500 g at birth)
- Very low birth weight (defined as weight < 1500 g at birth)
- High birth weight (defined as weight > 4000 g at birth)
- Large for gestational age (defined as birth weight > 90th centile for gestational age at delivery, based on standardised charts)
- Small for gestational age (defined as birth weight < 10th centile for gestational age at delivery, based on standardised charts)
- Congenital anomaly/birth defect (all congenital anomalies/birth defects identified will be included)
- Perinatal mortality (stillbirth or late and early neonatal deaths, up to 28 days after birth).

For neonatal secondary outcomes, where the unit of analysis is the baby, the method of analysis will account for the anticipated correlation in outcomes between multiple births.

### Pre-specified subgroup analysis

The consistency of the effect of type of embryo transfer across specific subgroups of couples will be assessed for the primary outcome using the statistical test of interaction, in addition to the adjusted model. Results will be presented on forest plots with adjusted risk ratios, 95% confidence intervals and the results of the interaction test.

Pre-specified subgroup analyses are:

- Fertility clinic
- Woman’s age (at the time of start of treatment i.e. ovarian stimulation): < 35 years, 35 to < 40 years, ≥ 40 years
- Blastocyst versus cleavage
- Single versus multiple embryo transfer
- Number of previous embryo transfers (0, 1–3, ≥ 4).

In addition, for those receiving frozen embryo transfer, the primary outcome will be summarised using numbers and percentages for the following subgroups:

- Natural versus hormone replacement cycles
- Vitrification versus slow freezing

### Sensitivity analysis

A CACE analysis will be conducted to assess the impact of non-compliance to the randomised allocation, i.e. women randomised to the frozen embryo transfer arm who receive fresh embryo transfer (non-compliers). This analytic technique provides a robust estimate of the treatment effect amongst compliant participants [11, 12].

The baseline characteristics of women randomised to the frozen embryo transfer arm will be reported by compliance status, and the unadjusted event rate for the primary outcome will be calculated for the observed compliers and non-compliers in the frozen embryo transfer arm. CACE analysis assumes that the proportion of would-be non-compliers in the fresh embryo transfer arm (i.e. couples in the fresh embryo transfer group who would not have complied had they been randomised to frozen embryo transfer) is the same as the proportion of non-compliers in the frozen embryo transfer group. It also assumes that the event rate among the non-compliers in the frozen embryo transfer arm is the same as the event rate among the would-be non-compliers in the fresh embryo transfer group. Applying these two assumptions, the unadjusted event rate for the primary outcome will be calculated for the would-be compliers and would-be non-compliers in the fresh embryo transfer arm. The unadjusted CACE risk ratio plus 95% confidence intervals for the primary outcome will be calculated using the event rates for compliant groups only (i.e. the observed compliers in the frozen embryo transfer arm and the would-be compliers in the fresh embryo transfer arm). The confidence intervals for the CACE estimated risk ratio will be calculated using bootstrapping methods [13].

If the entire record for a participant is considered fraudulent, this will be excluded from all analyses. If any individual data items are considered fraudulent, a sensitivity analysis will be conducted excluding these items.

### Significance levels and adjustment for multiplicity

The 95% confidence interval will be calculated for all analyses of the primary outcome. 99% confidence intervals will be used for all analyses of the secondary outcomes to take account of the number of hypothesis tests performed.

### Missing data

Missing data will be described by presenting the number of individuals in the missing category. All data collected on data collection forms will be used, since only essential data items will be collected.

For any partially completed self-evaluated STAI questionnaires in the Emotions Questionnaire CRF, if one or two items are omitted then the prorated full-scale score can be obtained by determining the mean weighted score for the scale items to which the individual responded, multiplying by 20 and rounding up to the next whole number. If three or more items are omitted then the full-scale score will be treated as missing [9].

### Statistical software employed

The statistical software Stata/SE version 15 (or later) will be used for all analyses.

## Safety data analysis

Serious adverse events will be listed by allocation.

## Additional exploratory analysis

The following exploratory analyses will be conducted on the primary outcome:

- A restricted per-protocol analysis, excluding couples who did not receive the allocated intervention as randomised
- An as-treated analysis, grouping couples according to the allocation they received

Analysis methods as used for the primary analysis will be employed.

Any other additional analyses not specified in the analysis plan will be exploratory in nature and a 1% two-sided significance level will be used with 99% confidence intervals. All such analyses will be approved by the Co-investigator Group.

## Deviation from analysis described in protocol

None yet.

## Supplementary information

**Additional file 1.** Appendix A – Detailed definition of outcomes v1.1.pdf.

**Additional file 2.** Appendix B – E-Freeze dummy tables v0.15.pdf.

**Additional file 3.** Appendix C – E-Freeze SAP v1.0.pdf.

## Data Availability

Information and files related to data management, the Trial Master File and Statistical Master File are held electronically on a secure server at the NPEU CTU.

## Abbreviations

AE: Adverse event
ANCOVA: Analysis of covariance
BMI: Body mass index
CACE: Complier-average causal effect
CI: Chief investigator
CONSORT: Consolidated standards of reporting trials
CRF: Case report form
CTU: Clinical Trials Unit
DMC: Data Monitoring Committee
eCRF: Electronic case report form
FSH: Follicle-stimulating hormone
g: Grams
GDM: Gestational diabetes mellitus
HFEA: Human Fertilisation and Embryology Authority
HTA: Health Technology Assessment
ICSI: Intracytoplasmic sperm injection
ITT: Intention to treat
IU: International units
IVF: In vitro fertilisation
kg: Kilogram
LMS: Lambda-mu-sigma
M: Metre
mm: Millimetre
NIHR: National Institute for Health Research
NNU: Neonatal unit
NPEU: National Perinatal Epidemiology Unit
OHSS: Ovarian hyperstimulation syndrome
RCOG: Royal College of Obstetricians and Gynaecologists
RCT: Randomised controlled trial
SAE: Serious adverse event
SAP: Statistical analysis plan
STAI: State-Trait Anxiety Inventory
TSC: Trail Steering Committee
UK: United Kingdom
