# Supplementary material for: E-Freeze - a randomised controlled trial evaluating the clinical and cost effectiveness of a policy of freezing embryos followed by thawed frozen embryo transfer compared with a policy of fresh embryo transfer, in women undergoing in vitro fertilisation: a statistical analysis plan
Source: Trials. 2020 Jun 30;21:596. doi: 10.1186/s13063-020-04441-9 (PMC7329511; doi:10.1186/s13063-020-04441-9)
Supplement: Supplementary file 1 — Additional file 1. Appendix A – Detailed definition of outcomes v1.1.pdf. [file 13063_2020_4441_MOESM1_ESM.pdf]

Appendix A: Detailed definition of outcomes

Outcome	Unit of analysis	Primary analysis denominator	Secondary analysis denominator (clinically relevant population)	Details of derivation
**Primary outcome**				
Healthy baby	Woman	ITT population		A healthy baby is defined as a live singleton baby born at term (between 37 and 42 completed weeks of gestation) with an appropriate weight for gestation (weight between 10th and 90th centile for that gestation based on standardised charts).<br><br>The LMS method [1] will be used to calculate birthweight centile.<br><br>Gestational age at birth is calculated as the difference between the expected date of delivery and 40 weeks, subtracted from the date of birth. This is divided by 7 to give the age in weeks.<br><br>Expected date of delivery is 38 weeks after egg collection for fresh transfer. For frozen transfer, the time between freezing and thawing is added on to this.
**Secondary outcomes**				
**Maternal safety outcome**				
Ovarian hyperstimulation syndrome (OHSS) - defined and classified as per the Royal College of Obstetricians and Gynaecologists (RCOG) green top guidelines	Woman	ITT population		
**Complications of pregnancy and delivery outcomes**				
Vanishing twin or triplet (defined as either: more fetal heartbeats than babies born, more gestational sacs than babies born, or more gestational sacs than fetal heartbeats)	Woman	ITT population	Total number of women with multiple gestational sacs and/or multiple fetal heartbeats	

Outcome	Unit of analysis	Primary analysis denominator	Secondary analysis denominator (clinically relevant population)	Details of derivation
Miscarriage rate (defined as pregnancy loss prior to age of viability i.e. 24 weeks of gestation)	Woman	ITT population	Total number of women with a positive pregnancy test at 2 weeks +/- 3 days after embryo transfer	Gestational age at a given date is calculated as the difference between the expected date of delivery and 40 weeks, subtracted from the given date. This is divided by 7 to give the age in weeks.<br><br>Expected date of delivery is 38 weeks after egg collection for fresh transfer. For frozen transfer, the time between freezing and thawing is added on to this.
Ectopic pregnancy	Woman	ITT population	Total number of women with a positive pregnancy test at 2 weeks +/- 3 days after embryo transfer	
Termination	Woman	ITT population	Total number of women with a positive pregnancy test at 2 weeks +/- 3 days after embryo transfer	
Gestational diabetes mellitus (GDM)	Woman	ITT population	Total number of pregnant women with an ongoing pregnancy resulting in delivery (live or stillbirth)	
Multiple pregnancy (defined as more than one fetal heartbeat or more than one gestational sac)	Woman	ITT population	Total number of women with a positive pregnancy test at 2 weeks +/- 3 days after embryo transfer	
Multiple births (including live and still births)	Woman	ITT population	Total number of pregnant women with an ongoing pregnancy resulting in delivery (live or stillbirth)	
Hypertensive disorders of pregnancy (chronic hypertension, pregnancy induced hypertension, pre-eclampsia, and eclampsia)	Woman	ITT population	Total number of pregnant women with an ongoing pregnancy resulting in delivery (live or stillbirth)	
Most severe hypertensive disorder (from least to worst: chronic	Woman	ITT population	Total number of pregnant women with an ongoing	

Outcome	Unit of analysis	Primary analysis denominator	Secondary analysis denominator (clinically relevant population)	Details of derivation
hypertension, pregnancy induced hypertension, pre-eclampsia, and eclampsia)			pregnancy resulting in delivery (live or stillbirth)	
Antepartum haemorrhage (any bleeding per vaginum after 28 weeks of pregnancy including placenta praevia and placental abruption)	Woman	ITT population	Total number of pregnant women with an ongoing pregnancy resulting in delivery (live or stillbirth)	
Onset of labour (spontaneous, induced or planned caesarean section)	Woman	ITT population	Total number of pregnant women with an ongoing pregnancy resulting in delivery (live or stillbirth)	
Mode of delivery for each baby (normal vaginal delivery, instrumental vaginal delivery or caesarean section)	Woman	ITT population	Total number of infants of pregnant women with an ongoing pregnancy resulting in delivery (live or stillbirth)	
Preterm delivery (defined as delivery at < 37 completed weeks)	Woman	ITT population	Total number of pregnant women with an ongoing pregnancy resulting in delivery (live or stillbirth)	Gestational age at birth is calculated as the difference between the expected date of delivery and 40 weeks, subtracted from the date of birth. This is divided by 7 to give the age in weeks.<br><br>Expected date of delivery is 38 weeks after egg collection for fresh transfer. For frozen transfer, the time between freezing and thawing is added on to this.
Very preterm delivery (defined as delivery at < 32 completed weeks)	Woman	ITT population	Total number of pregnant women with an ongoing pregnancy resulting in delivery (live or stillbirth)	Gestational age at birth is calculated as the difference between the expected date of delivery and 40 weeks, subtracted from the date of birth. This is divided by 7 to give the age in weeks.<br><br>Expected date of delivery is 38 weeks after egg collection for fresh transfer. For frozen transfer, the time between freezing and thawing is added on to this.

Outcome	Unit of analysis	Primary analysis denominator	Secondary analysis denominator (clinically relevant population)	Details of derivation
Low birth weight (defined as weight < 2500g at birth)	Infant	ITT population	Total number of babies born	
Very low birth weight (defined as weight < 1500g at birth)	Infant	ITT population	Total number of babies born	
High birth weight (defined as weight > 4000g at birth)	Infant	ITT population	Total number of babies born	
Large for gestational age (defined as birth weight > 90th centile for gestational age at delivery, based on standardised charts)	Infant	ITT population	Total number of babies born	Using LMS method for birthweight centiles [1].<br><br>Gestational age at birth is calculated as the difference between the expected date of delivery and 40 weeks, subtracted from the date of birth. This is divided by 7 to give the age in weeks.<br><br>Expected date of delivery is 38 weeks after egg collection for fresh transfer. For frozen transfer, the time between freezing and thawing is added on to this.
Small for gestational age (defined as birth weight < 10th centile for gestational age at delivery, based on standardised charts)	Infant	ITT population	Total number of babies born	Using LMS method for birthweight centiles [1].<br><br>Gestational age at birth is calculated as the difference between the expected date of delivery and 40 weeks, subtracted from the date of birth. This is divided by 7 to give the age in weeks.<br><br>Expected date of delivery is 38 weeks after egg collection for fresh transfer. For frozen transfer, the time between freezing and thawing is added on to this.
Congenital anomaly/birth defect (all congenital anomalies/birth defects identified will be included)	Infant	ITT population	Total number of babies born	

Outcome	Unit of analysis	Primary analysis denominator	Secondary analysis denominator (clinically relevant population)	Details of derivation
Perinatal mortality (stillbirth or late as well as early neonatal deaths, up to 28 days after birth)	Infant	ITT population	Total number of babies born	
**Measures of clinical effectiveness outcomes**				
Live birth rate (this is a live birth episode i.e. twins will count as one)	Woman	ITT population		
Singleton live birth rate	Woman	ITT population		
Singleton live birth rate at term	Woman	ITT population		Gestational age at birth is calculated as the difference between the expected date of delivery and 40 weeks, subtracted from the date of birth. This is divided by 7 to give the age in weeks.<br><br>Expected date of delivery is 38 weeks after egg collection for fresh transfer. For frozen transfer, the time between freezing and thawing is added on to this.
Singleton baby with appropriate weight for gestation	Woman	ITT population		The LMS method will be used to calculate birthweight centiles, which will be between the 10th and 90th centile.<br><br>Gestational age at birth is calculated as the difference between the expected date of delivery and 40 weeks, subtracted from the date of birth. This is divided by 7 to give the age in weeks.<br><br>Expected date of delivery is 38 weeks after egg collection for fresh transfer. For frozen transfer, the time between freezing and thawing is added on to this.
Pregnancy rate (defined as positive pregnancy test at 2 weeks +/- 3 days after embryo transfer)	Woman	ITT population		
Clinical pregnancy rate (defined as the presence of at least one fetal heartbeat at ultrasound between six	Woman	ITT population		

Outcome	Unit of analysis	Primary analysis denominator	Secondary analysis denominator (clinically relevant population)	Details of derivation
and eight weeks gestation; ectopic pregnancy counts as a clinical pregnancy; multiple gestational sacs count as one clinical pregnancy)				
**Measures of the effectiveness of the process of freezing embryos outcomes**				
Total number of embryos frozen, thawed and transferred for all randomised couples	Couple	Total number of randomised couples		
Proportion of thawed embryos that were then transferred for all randomised couples	Couple	Total number of randomised couples		
Failure of all embryos to survive after thawing leading to no embryo transfer	Couple	ITT population		
**Other secondary outcomes**				
Evaluation of emotional state (for both the female and male partners)	Man<br>Woman	Total number of randomised couples		

References

1. Cole TJ, Freeman JV, Preece MA. British 1990 growth reference centiles for weight, height, body mass index and head circumference fitted by maximum penalized likelihood. *Stat Med* 1998; 17(4): 407-29.
