# Supplementary material for: E-Freeze - a randomised controlled trial evaluating the clinical and cost effectiveness of a policy of freezing embryos followed by thawed frozen embryo transfer compared with a policy of fresh embryo transfer, in women undergoing in vitro fertilisation: a statistical analysis plan
Source: Trials. 2020 Jun 30;21:596. doi: 10.1186/s13063-020-04441-9 (PMC7329511; doi:10.1186/s13063-020-04441-9)
Supplement: Supplementary file 2 — Additional file 2. Appendix B – E-Freeze dummy tables v0.15.pdf. [file 13063_2020_4441_MOESM2_ESM.pdf]

E-Freeze

Full title: Elective Freezing of embryos in assisted conception: a randomised controlled trial evaluating the clinical and cost-effectiveness of a policy of freezing embryos followed by thawed frozen embryo transfer, compared with a policy of fresh embryo transfer in women undergoing in vitro fertilisation

ISRCTN61225414
REC reference: 15/NS/0114

Dummy tables

Version 0.15, 19 May 2020

Authors:
Jennifer Bell (E-Freeze Trial statistician 2017 to present, NPEU CTU)
Melanie Greenland (E-Freeze Trial statistician 2015 to 2017, NPEU CTU)

Reviewers:
Dr Abha Maheshwari (Chief Investigator)
Pollyanna Hardy (Senior statistician 2014 to 2017, NPEU CTU)
Dr Louise Linsell (Senior statistician 2017 to present, NPEU CTU)
Ed Juszczak (NPEU CTU Director)

NPEU Clinical Trials Unit

UKCRC Registered Clinical Trials Units

UNIVERSITY OF OXFORD

Contents

List of abbreviations 3

Figure 1: Flow of participants 4

Primary analysis 5

Table 1: Demographic and clinical characteristics at trial entry 6

Table 2: Clinical characteristics of embryo and endometrium 10

Table 3: Primary outcome 12

Table 4a: Secondary outcomes – maternal safety 13

Table 4b: Secondary outcomes – Complications of pregnancy and delivery 14

Table 4c: Secondary outcomes – Measures of clinical effectiveness 17

Table 5a: Secondary outcomes – measures of effectiveness of the process of freezing embryos 18

Table 5b: Secondary outcomes – measures of effectiveness of the process of freezing embryos (continued) 19

Table 6: Secondary outcomes – evaluation of emotional state 20

Table 7: Adherence 22

Table 8: Unexpected serious adverse events by allocation 23

Table 9: Protocol non-compliances 24

Secondary analyses 25

Table 10: Clinically important outcomes 26

Table 11: Subgroup analyses for primary outcome 29

Table 12: Exploratory analysis 30

List of abbreviations

AE	adverse event
BMI	body mass index
CACE	complier average causal effect
CI	confidence interval
cm	centimetre
CTU	clinical trials unit
DHEA	dehydroepiandrosterone/dehydroepiandrostenedione
EPS	early pregnancy scan
FSH	follicle-stimulating hormone
g	gram
GDM	gestational diabetes mellitus
HCG	human chorionic gonadotropin
ICSI	intracytoplasmic sperm injection
IQR	interquartile range
IVF	in vitro fertilisation
kg	kilogram
m	metre
max	maximum
MD	mean difference
Med	median
min	minimum
mm	millimetre
NPEU	National Perinatal Epidemiology Unit
OHSS	ovarian hyperstimulation syndrome
PE	point estimate
RR	risk ratio
SAE	serious adverse event
SD	standard deviation

Figure 1: Flow of participants

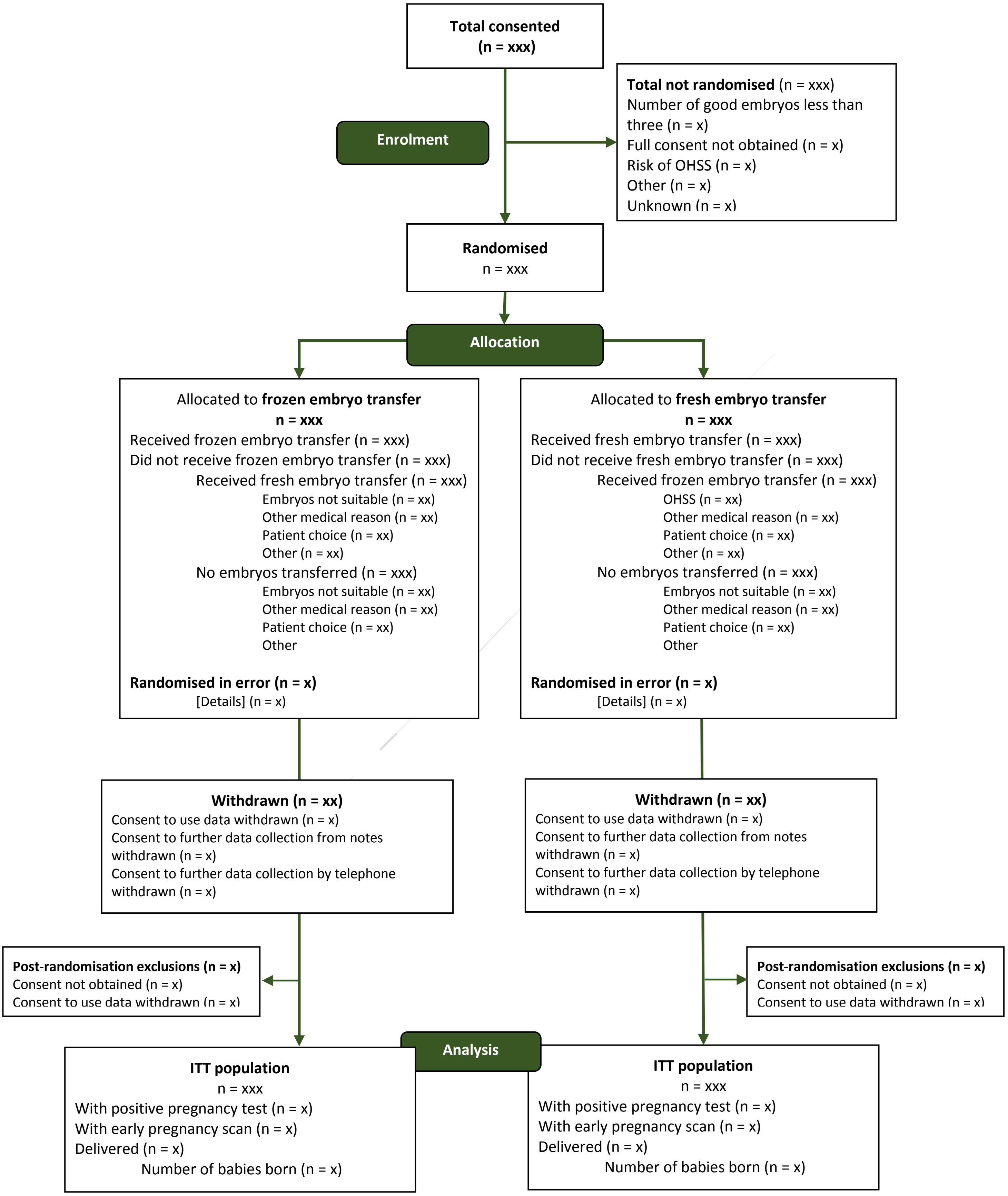

Primary analysis

Table 1: Demographic and clinical characteristics at trial entry

		Frozen embryo transfer (n=XXX)	Fresh embryo transfer (n=XXX)
**Fertility clinic[1]**			
1	n (%)	xx (xx.x)	xx (xx.x)
2	n (%)	xx (xx.x)	xx (xx.x)
3	n (%)	xx (xx.x)	xx (xx.x)
4	n (%)	xx (xx.x)	xx (xx.x)
5	n (%)	xx (xx.x)	xx (xx.x)
…		…	
**Woman's age at ovarian stimulation[1] (years)**	Mean {SD}	xx.x (x.xx)	xx.x (x.xx)
	Med [IQR]	xx [xx to xx]	xx [xx to xx]
	(Min to max)	xx (xx to xx)	xx (xx to xx)
< 35	n (%)	xx (xx.x)	xx (xx.x)
35 to < 40	n (%)	xx (xx.x)	xx (xx.x)
≥ 40	n (%)	xx (xx.x)	xx (xx.x)
**Woman's ethnicity[2]**			
White	n (%)	xx (xx.x)	xx (xx.x)
Black	n (%)	xx (xx.x)	xx (xx.x)
Asian	n (%)	xx (xx.x)	xx (xx.x)
Mixed	n (%)	xx (xx.x)	xx (xx.x)
Other	n (%)	xx (xx.x)	xx (xx.x)
Unknown	n	xx	xx
Missing	n	xx	xx
**Woman's smoking status**			
Never smoked	n (%)	xx (xx.x)	xx (xx.x)
Past smoker	n (%)	xx (xx.x)	xx (xx.x)
Current smoker	n (%)	xx (xx.x)	xx (xx.x)
Missing	n	xx	xx
**Woman's BMI (kg/m²)**	Mean {SD}	xx.x (x.xx)	xx.x (x.xx)
	Med [IQR]	xx [xx to xx]	xx [xx to xx]
	(Min to max)	xx (xx to xx)	xx (xx to xx)
Missing	n	xx	xx
Underweight (< 18.5)	n (%)	xx (xx.x)	xx (xx.x)
Healthy weight (18.5 to 24.9)	n (%)	xx (xx.x)	xx (xx.x)
Overweight (25 to 29.9)	n (%)	xx (xx.x)	xx (xx.x)

[1] Minimisation criteria
[2] Added to eCRFs part-way through the trial on 12 April 2017, resulting in missing data for couples recruited prior to that date

		Frozen embryo transfer (n=XXX)	Fresh embryo transfer (n=XXX)
Obese (30 to 34.9)	n (%)	xx (xx.x)	xx (xx.x)
Very obese (> 35)	n (%)	xx (xx.x)	xx (xx.x)
**Type of infertility**[3]			
Primary	n (%)	xx (xx.x)	xx (xx.x)
Secondary	n (%)	xx (xx.x)	xx (xx.x)
**Woman's previous pregnancies**			
0	n (%)	xx (xx.x)	xx (xx.x)
1	n (%)	xx (xx.x)	xx (xx.x)
2	n (%)	xx (xx.x)	xx (xx.x)
>2	n (%)	xx (xx.x)	xx (xx.x)
Missing	n	xx	xx
**Woman's previous live births**			
0	n (%)	xx (xx.x)	xx (xx.x)
1	n (%)	xx (xx.x)	xx (xx.x)
2	n (%)	xx (xx.x)	xx (xx.x)
>2	n (%)	xx (xx.x)	xx (xx.x)
Missing	n	xx	xx
**Main cause of infertility**			
Ovulatory	n (%)	xx (xx.x)	xx (xx.x)
Tubal	n (%)	xx (xx.x)	xx (xx.x)
Endometriosis	n (%)	xx (xx.x)	xx (xx.x)
Unexplained	n (%)	xx (xx.x)	xx (xx.x)
Male	n (%)	xx (xx.x)	xx (xx.x)
Other	n (%)	xx (xx.x)	xx (xx.x)
Missing	n	xx	xx
**Duration of infertility**[3] **(months)**	Mean {SD}<br>Med [IQR]<br>(Min to max)	xx.x (x.xx)<br>xx [xx to xx]<br>xx (xx to xx)	xx.x (x.xx)<br>xx [xx to xx]<br>xx (xx to xx)
< 12	n (%)	xx (xx.x)	xx (xx.x)
12 to < 24	n (%)	xx (xx.x)	xx (xx.x)
24 to < 36	n (%)	xx (xx.x)	xx (xx.x)
36 to < 48	n (%)	xx (xx.x)	xx (xx.x)
48 to < 60	n (%)	xx (xx.x)	xx (xx.x)
≥ 60	n (%)	xx (xx.x)	xx (xx.x)
**Endoscratch performed**	n (%)	xx (xx.x)	xx (xx.x)
Missing	n	xx	xx
**Stimulation regimen used**			

[3] Minimisation criteria

		Frozen embryo transfer (n=XXX)	Fresh embryo transfer (n=XXX)
Long	n (%)	xx (xx.x)	xx (xx.x)
Short	n (%)	xx (xx.x)	xx (xx.x)
Ultrashort	n (%)	xx (xx.x)	xx (xx.x)
Antagonist	n (%)	xx (xx.x)	xx (xx.x)
Other	n (%)	xx (xx.x)	xx (xx.x)
Missing	n	xx	xx
Missing	n	xx	xx
**Total stimulation dose of FSH (IU)**	Mean {SD}	xx.x (x.xx)	xx.x (x.xx)
	Med [IQR]	xx [xx to xx]	xx [xx to xx]
	(Min to max)	xx (xx to xx)	xx (xx to xx)
Missing	n	xx	xx
**Adjuvants used (non-exclusive)**	n (%)	xx (xx.x)	xx (xx.x)
Aspirin	n (%)	xx (xx.x)	xx (xx.x)
Heparin	n (%)	xx (xx.x)	xx (xx.x)
Steroids	n (%)	xx (xx.x)	xx (xx.x)
Growth hormone	n (%)	xx (xx.x)	xx (xx.x)
DHEA	n (%)	xx (xx.x)	xx (xx.x)
Missing	n	xx	xx
Missing	n	xx	xx
**Blood test performed on day of trigger injection**	n (%)	xx (xx.x)	xx (xx.x)
Missing	n	xx	xx
**Trigger injection used**			
Agonist	n (%)	xx (xx.x)	xx (xx.x)
Dual trigger	n (%)	xx (xx.x)	xx (xx.x)
HCG	n (%)	xx (xx.x)	xx (xx.x)
Missing	n	xx	xx
**Total number of eggs collected**	Mean {SD}	xx.x (x.xx)	xx.x (x.xx)
	Med [IQR]	xx [xx to xx]	xx [xx to xx]
	(Min to max)	xx (xx to xx)	xx (xx to xx)
Missing	n	xx	xx
3 to 5	n (%)	xx (xx.x)	xx (xx.x)
6 to 9	n (%)	xx (xx.x)	xx (xx.x)
10 to 15	n (%)	xx (xx.x)	xx (xx.x)
> 15	n (%)	xx (xx.x)	xx (xx.x)
Missing	n	xx	xx
**Method of insemination**[4]			
IVF	n (%)	xx (xx.x)	xx (xx.x)

[4] Minimisation criteria

		Frozen embryo transfer (n=XXX)	Fresh embryo transfer (n=XXX)
ICSI	n (%)	xx (xx.x)	xx (xx.x)
Split (IVF & ICSI)	n (%)	xx (xx.x)	xx (xx.x)
**Number of eggs fertilised normally (two pro nucleus)**	Mean (SD)	xx.x (x.xx)	xx.x (x.xx)
	Med [IQR]	xx [xx to xx]	xx [xx to xx]
	(Min to max)	xx (xx to xx)	xx (xx to xx)
Missing	n	xx	xx
3 to 5	n (%)	xx (xx.x)	xx (xx.x)
6 to 9	n (%)	xx (xx.x)	xx (xx.x)
10 to 15	n (%)	xx (xx.x)	xx (xx.x)
> 15	n (%)	xx (xx.x)	xx (xx.x)
Missing	n	xx	xx
**Time lapse used**	n (%)	xx (xx.x)	xx (xx.x)
Missing	n	xx	xx
**Good quality embryos created on day three**	Mean {SD}	xx.x (x.xx)	xx.x (x.xx)
	Med [IQR]	xx [xx to xx]	xx [xx to xx]
	(Min to max)	xx (xx to xx)	xx (xx to xx)
Missing	n	xx	xx
3 to 4	n (%)	xx (xx.x)	xx (xx.x)
5 to 6	n (%)	xx (xx.x)	xx (xx.x)
7 to 10	n (%)	xx (xx.x)	xx (xx.x)
> 10	n (%)	xx (xx.x)	xx (xx.x)
**Number of previous egg collections**[5]			
0	n (%)	xx (xx.x)	xx (xx.x)
1	n (%)	xx (xx.x)	xx (xx.x)
2	n (%)	xx (xx.x)	xx (xx.x)
≥ 3	n (%)	xx (xx.x)	xx (xx.x)
Missing	n	xx	xx
**Number of previous embryo transfers**			
0	n (%)	xx (xx.x)	xx (xx.x)
1 to 3	n (%)	xx (xx.x)	xx (xx.x)
≥ 4	n (%)	xx (xx.x)	xx (xx.x)
Missing	n	xx	xx

[5] Minimisation criteria

Table 2: Clinical characteristics of embryo and endometrium

		Frozen embryo transfer (n=XXX)	Fresh embryo transfer (n=XXX)
**No embryo transfer**	n	xxx	xxx
**Had embryo transfer**	**N**	XXX	XXX
**Stage of embryo at transfer**			
Cleavage (day 3)	n (%)	xx (xx.x)	xx (xx.x)
Cleavage (day 4)	n (%)	xx (xx.x)	xx (xx.x)
Blastocyst (day 5)	n (%)	xx (xx.x)	xx (xx.x)
Blastocyst (day 6)	n (%)	xx (xx.x)	xx (xx.x)
Missing	n	xx	xx
**Number of embryos transferred**	Mean {SD}	xx.x (x.xx)	xx.x (x.xx)
	Med [IQR]	xx [xx to xx]	xx [xx to xx]
	(Min to max)	xx (xx to xx)	xx (xx to xx)
Missing	n	xx	xx
1	n (%)	xx (xx.x)	xx (xx.x)
2	n (%)	xx (xx.x)	xx (xx.x)
3	n (%)	xx (xx.x)	xx (xx.x)
**Number of remaining frozen embryos after transfer**[6]	Mean {SD}	xx.x (x.xx)	xx.x (x.xx)
	Med [IQR]	xx [xx to xx]	xx [xx to xx]
	(Min to max)	xx (xx to xx)	xx (xx to xx)
Missing	n	xx	xx
0	n (%)	xx (xx.x)	xx (xx.x)
1	n (%)	xx (xx.x)	xx (xx.x)
2	n (%)	xx (xx.x)	xx (xx.x)
> 2	n (%)	xx (xx.x)	xx (xx.x)
**Endometrial appearance**			
Triple layer	n (%)	xx (xx.x)	xx (xx.x)
No triple layer	n (%)	xx (xx.x)	xx (xx.x)
Missing	n	xx	xx
**Endometrial thickness (mm)**	Mean {SD}	xx.x (x.xx)	xx.x (x.xx)
	Med [IQR]	xx [xx to xx]	xx [xx to xx]
	(Min to max)	xx (xx to xx)	xx (xx to xx)
Not recorded	n	xx	xx

[6] For fresh embryo transfer the remaining embryos not transferred are then frozen for future cycles. For frozen embryo transfer the remaining embryos that are not thawed remain frozen.

		Frozen embryo transfer (n=XXX)	Fresh embryo transfer (n=XXX)
**Received frozen transfer**	**N**	XXX	XXX
**Method of embryo freezing**			
Vitrification	n (%)	xx (xx.x)	xx (xx.x)
Slow freezing	n (%)	xx (xx.x)	xx (xx.x)
Missing	n	xx	xx
**Number of embryos frozen**	Mean {SD}	xx.x (x.xx)	xx.x (x.xx)
	Med [IQR]	xx [xx to xx]	xx [xx to xx]
	(Min to max)	xx (xx to xx)	xx (xx to xx)
Missing	n	xx	xx
1	n (%)	xx (xx.x)	xx (xx.x)
2	n (%)	xx (xx.x)	xx (xx.x)
> 2	n (%)	xx (xx.x)	xx (xx.x)
**Number of embryos thawed**	Mean {SD}	xx.x (x.xx)	xx.x (x.xx)
	Med [IQR]	xx [xx to xx]	xx [xx to xx]
	(Min to max)	xx (xx to xx)	xx (xx to xx)
Missing	n	xx	xx
1	n (%)	xx (xx.x)	xx (xx.x)
2	n (%)	xx (xx.x)	xx (xx.x)
> 2	n (%)	xx (xx.x)	xx (xx.x)
**Method of endometrial preparation for transfer**			
Natural cycle	n (%)	xx (xx.x)	xx (xx.x)
Natural cycle with HCG	n (%)	xx (xx.x)	xx (xx.x)
Artificial cycle with estrogen and progesterone	n (%)	xx (xx.x)	xx (xx.x)
Artificial cycle with estrogen and progesterone and down regulation with GnRH agonist (buserelin)	n (%)	xx (xx.x)	xx (xx.x)
Other	n (%)	xx (xx.x)	xx (xx.x)
Missing	n	xx	xx
**Time from egg collection to embryo freezing**			
Cleavage (day 3)	n (%)	xx (xx.x)	xx (xx.x)
Cleavage (day 4)	n (%)	xx (xx.x)	xx (xx.x)
Blastocyst (day 5)	n (%)	xx (xx.x)	xx (xx.x)
Blastocyst (day 6)	n (%)	xx (xx.x)	xx (xx.x)
< 3 days	n (%)	xx (xx.x)	xx (xx.x)
> 6 days	n (%)	xx (xx.x)	xx (xx.x)
Missing	n	xx	xx

Table 3: Primary outcome

		Frozen embryo transfer (n=XXX)	Fresh embryo transfer (n=XXX)	Risk ratio (95% CI)
**Singleton baby born at term with appropriate weight for gestation**	n (%)	xx (xx.x)	xx (xx.x)	RR (xx.x to xx.x)
**Did not have primary outcome**	n (%)	xx (xx.x)	xx (xx.x)	
No embryo transfer	n (%)	xx (xx.x)	xx (xx.x)	
Negative pregnancy test	n (%)	xx (xx.x)	xx (xx.x)	
Loss of pregnancy[7]	n (%)	xx (xx.x)	xx (xx.x)	
Other delivery outcome	n (%)	xx (xx.x)	xx (xx.x)	
Missing	n	xx	xx	

[7] Miscarriage, ectopic, pregnancy of unknown location, or termination

Table 4a: Secondary outcomes – maternal safety

		Frozen embryo transfer (n=XXX)	Fresh embryo transfer (n=XXX)	Effect estimate (99% CI)
**Ovarian hyperstimulation syndrome (OHSS)**	n (%)	xx (xx.x)	xx (xx.x)	RR (xx.x to xx.x)
Mild	n (%)	xx (xx.x)	xx (xx.x)	
Moderate	n (%)	xx (xx.x)	xx (xx.x)	
Severe	n (%)	xx (xx.x)	xx (xx.x)	
Missing	n	xx	xx	
Missing	n	xx	xx	

Table 4b: Secondary outcomes – Complications of pregnancy and delivery

		Frozen embryo transfer (n=XXX)	Fresh embryo transfer (n=XXX)	Effect estimate (99% CI)
**Vanishing twin/triplet**	n (%)	xx (xx.x)	xx (xx.x)	RR (xx.x to xx.x)
Missing	n	xx	xx	
**Pregnancy loss**				
Miscarriage	n (%)	xx (xx.x)	xx (xx.x)	RR (xx.x to xx.x)
Early (< 12 weeks' gestation)	n (%)	xx (xx.x)	xx (xx.x)	
Late (12 to < 24 weeks' gestation)	n (%)	xx (xx.x)	xx (xx.x)	
Gestation unknown	n	xx	xx	
Ectopic	n (%)	xx (xx.x)	xx (xx.x)	RR (xx.x to xx.x)
Termination	n (%)	xx (xx.x)	xx (xx.x)	RR (xx.x to xx.x)
Pregnancy of unknown location (PUL)	n (%)	xx (xx.x)	xx (xx.x)	
Missing	n	xx	xx	
**Gestational diabetes mellitus (GDM)**	n (%)	xx (xx.x)	xx (xx.x)	RR (xx.x to xx.x)
Missing	n	xx	xx	
**Multiple pregnancy**	n (%)	xx (xx.x)	xx (xx.x)	RR (xx.x to xx.x)
Missing	n	xx	xx	
**Multiple births**	n (%)	xx (xx.x)	xx (xx.x)	RR (xx.x to xx.x)
Missing	n	xx	xx	
**Hypertensive disorder**	n (%)	xx (xx.x)	xx (xx.x)	RR (xx.x to xx.x)
Chronic hypertension	n (%)	xx (xx.x)	xx (xx.x)	
Pregnancy induced hypertension	n (%)	xx (xx.x)	xx (xx.x)	
Pre-eclampsia	n (%)	xx (xx.x)	xx (xx.x)	
Eclampsia	n (%)	xx (xx.x)	xx (xx.x)	
Missing	n	xx	xx	
**Most severe hypertensive disorder**				
Chronic hypertension	n (%)	xx (xx.x)	xx (xx.x)	
Pregnancy induced hypertension	n (%)	xx (xx.x)	xx (xx.x)	
Pre-eclampsia	n (%)	xx (xx.x)	xx (xx.x)	
Eclampsia	n (%)	xx (xx.x)	xx (xx.x)	
Missing	n	xx	xx	
**Antepartum haemorrhage (non-exclusive)**				RR (xx.x to xx.x)
Placenta praevia	n (%)	xx (xx.x)	xx (xx.x)	
Placental abruption	n (%)	xx (xx.x)	xx (xx.x)	

		Frozen embryo transfer (n=XXX)	Fresh embryo transfer (n=XXX)	Effect estimate (99% CI)
Other	n (%)	xx (xx.x)	xx (xx.x)	
Missing	n	xx	xx	
Missing	n	xx	xx	
**Onset of labour**				
Spontaneous	n (%)	xx (xx.x)	xx (xx.x)	RR (xx.x to xx.x)
Induced	n (%)	xx (xx.x)	xx (xx.x)	
Planned caesarean section	n (%)	xx (xx.x)	xx (xx.x)	
Missing	n	xx	xx	
**Mode of delivery**				
Normal vaginal delivery	n (%)	xx (xx.x)	xx (xx.x)	RR (xx.x to xx.x)
Instrumental vaginal delivery	n (%)	xx (xx.x)	xx (xx.x)	RR (xx.x to xx.x)
Caesarean section	n (%)	xx (xx.x)	xx (xx.x)	RR (xx.x to xx.x)
Missing	n	xx	xx	
**Preterm delivery (<37 completed weeks)**	n (%)	xx (xx.x)	xx (xx.x)	RR (xx.x to xx.x)
Missing	n	xx	xx	
**Very preterm delivery (<32 completed weeks)**	n (%)	xx (xx.x)	xx (xx.x)	RR (xx.x to xx.x)
Missing	n	xx	xx	
**Low birth weight (<2500 g at birth)**	n (%)	xx (xx.x)	xx (xx.x)	RR (xx.x to xx.x)
Missing	n	xx	xx	
**Very low birth weight (<1500 g at birth)**	n (%)	xx (xx.x)	xx (xx.x)	RR (xx.x to xx.x)
Missing	n	xx	xx	
**High birth weight (>4000 g at birth)**	n (%)	xx (xx.x)	xx (xx.x)	RR (xx.x to xx.x)
Missing	n	xx	xx	
**Customised birth weight centile**[8]	Mean {SD}<br>Med [IQR]<br>(Min to max)	xx.x (x.xx)<br>xx [xx to xx]<br>xx (xx to xx)	xx.x (x.xx)<br>xx [xx to xx]<br>xx (xx to xx)	
Missing	n	xx	xx	
**Large for gestational age (>90th centile)**	n (%)	xx (xx.x)	xx (xx.x)	RR (xx.x to xx.x)
Missing	n	xx	xx	

[8] Cole TJ, Freeman JV, Preece MA. British 1990 growth reference centiles for weight, height, body mass index and head circumference fitted by maximum penalized likelihood. Stat Med 1998; 17(4):407-29

		Frozen embryo transfer (n=XXX)	Fresh embryo transfer (n=XXX)	Effect estimate (99% CI)
**Small for gestational age (<10th centile)**	n (%)	xx (xx.x)	xx (xx.x)	RR (xx.x to xx.x)
Missing	n	xx	xx	
**Congenital anomaly/birth defect**	n (%)	xx (xx.x)	xx (xx.x)	RR (xx.x to xx.x)
Missing	n	xx	xx	
**Perinatal mortality up to 28 days after birth**	n (%)	xx (xx.x)	xx (xx.x)	RR (xx.x to xx.x)
Stillbirth	n (%)	xx (xx.x)	xx (xx.x)	
Neonatal death up to 28 days after birth	n (%)	xx (xx.x)	xx (xx.x)	
Missing	n	xx	xx	

Table 4c: Secondary outcomes – Measures of clinical effectiveness

		Frozen embryo transfer (n=XXX)	Fresh embryo transfer (n=XXX)	Effect estimate (99% CI)
**Live birth episode**	n (%)	xx (xx.x)	xx (xx.x)	RR (xx.x to xx.x)
Missing	n	xx	xx	
**Singleton baby**	n (%)	xx (xx.x)	xx (xx.x)	RR (xx.x to xx.x)
Missing	n	xx	xx	
**Singleton baby born at term**	n (%)	xx (xx.x)	xx (xx.x)	RR (xx.x to xx.x)
Missing	n	xx	xx	
**Singleton baby with appropriate weight for gestation**	n (%)	xx (xx.x)	xx (xx.x)	
Missing	n	xx	xx	
**Pregnancy test at two weeks after embryo transfer**				
Positive	n (%)	xx (xx.x)	xx (xx.x)	RR (xx.x to xx.x)
Negative	n (%)	xx (xx.x)	xx (xx.x)	
Missing	n	xx	xx	
**Clinical pregnancy**	n (%)	xx (xx.x)	xx (xx.x)	RR (xx.x to xx.x)
Missing	n	xx	xx	
EPS performed	n (%)	xx (xx.x)	xx (xx.x)	
Ongoing	n (%)	xx (xx.x)	xx (xx.x)	
Ectopic	n (%)	xx (xx.x)	xx (xx.x)	
Pregnancy of unknown location	n (%)	xx (xx.x)	xx (xx.x)	
Miscarriage	n (%)	xx (xx.x)	xx (xx.x)	
Missing	n	xx	xx	
EPS not performed	n (%)	xx (xx.x)	xx (xx.x)	
Moved away from UK	n (%)	xx (xx.x)	xx (xx.x)	
Pregnancy lost before date of scan	n (%)	xx (xx.x)	xx (xx.x)	
No embryo transfer, or negative pregnancy test	n (%)	xx (xx.x)	xx (xx.x)	
Other	n (%)	xx (xx.x)	xx (xx.x)	
Missing	n	xx	xx	
Missing	n	xx	xx	

Table 5a: Secondary outcomes – measures of effectiveness of the process of freezing embryos

		All couples (n=XXX)
**Total number of embryos**		
Frozen	n (%)	xx (xx.x)
Thawed	n (%)	xx (xx.x)
Transferred	n (%)	xx (xx.x)
**Thawed embryos that were then transferred**	n (%)	xx (xx.x)
Missing couples	n	xx

Table 5b: Secondary outcomes – measures of effectiveness of the process of freezing embryos (continued)

		Frozen embryo transfer (n=XXX)	Fresh embryo transfer (n=XXX)
**Failure of all embryos to survive after thawing**	n (%)	xx (xx.x)	xx (xx.x)
Missing	n	xx	xx

Table 6: Secondary outcomes – evaluation of emotional state

		Frozen embryo transfer (n=XXX)	Fresh embryo transfer (n=XXX)	Effect estimate (99% CI)
**State scores at randomisation**				
Female partner's STAI score[9]	Mean {SD}	xx.x (x.xx)	xx.x (x.xx)	
	Med [IQR]	xx [xx to xx]	xx [xx to xx]	
	(Min to max)	xx (xx to xx)	xx (xx to xx)	
Missing	n	xx	xx	
Male partner's STAI score	Mean {SD}	xx.x (x.xx)	xx.x (x.xx)	
	Med [IQR]	xx [xx to xx]	xx [xx to xx]	
	(Min to max)	xx (xx to xx)	xx (xx to xx)	
Missing	n	xx	xx	
**Satisfaction with IVF treatment process at randomisation**				
Female partner's overall satisfaction with the process of IVF treatment so far				
Very satisfied	n (%)	xx (xx.x)	xx (xx.x)	
Satisfied	n (%)	xx (xx.x)	xx (xx.x)	
Neither satisfied nor dissatisfied	n (%)	xx (xx.x)	xx (xx.x)	
Dissatisfied	n (%)	xx (xx.x)	xx (xx.x)	
Very dissatisfied	n (%)	xx (xx.x)	xx (xx.x)	
Missing	n	xx	xx	
Male partner's overall satisfaction with the process of IVF treatment so far at randomisation				
Very satisfied	n (%)	xx (xx.x)	xx (xx.x)	
Satisfied	n (%)	xx (xx.x)	xx (xx.x)	
Neither satisfied nor dissatisfied	n (%)	xx (xx.x)	xx (xx.x)	
Dissatisfied	n (%)	xx (xx.x)	xx (xx.x)	
Very dissatisfied	n (%)	xx (xx.x)	xx (xx.x)	
Missing	n	xx	xx	
**State scores post embryo transfer**				
Female partner's STAI score	Mean {SD}	xx.x (x.xx)	xx.x (x.xx)	MD (xx.x to xx.x)
	Med [IQR]	xx [xx to xx]	xx [xx to xx]	
	(Min to max)	xx (xx to xx)	xx (xx to xx)	

[9] Spielberger, C. D., Gorsuch, R. L., Lushene, R., Vagg, P. R., & Jacobs, G. A. (1983). *Manual for the State-Trait Anxiety Inventory*. Palo Alto, CA: Consulting Psychologists Press.

		Frozen embryo transfer (n=XXX)	Fresh embryo transfer (n=XXX)	Effect estimate (99% CI)
Missing	n	xx	xx	
Male partner's STAI score	Mean {SD}<br>Med [IQR]<br>(Min to max)	xx.x (x.xx)<br>xx [xx to xx]<br>xx (xx to xx)	xx.x (x.xx)<br>xx [xx to xx]<br>xx (xx to xx)	MD (xx.x to xx.x)
Missing	n	xx	xx	
**Satisfaction with IVF treatment process post embryo transfer**				
Female partner's overall satisfaction with the process of IVF treatment so far				
Very satisfied	n (%)	xx (xx.x)	xx (xx.x)	
Satisfied	n (%)	xx (xx.x)	xx (xx.x)	
Neither satisfied nor dissatisfied	n (%)	xx (xx.x)	xx (xx.x)	
Dissatisfied	n (%)	xx (xx.x)	xx (xx.x)	
Very dissatisfied	n (%)	xx (xx.x)	xx (xx.x)	
Missing	n	xx	xx	
Male partner's overall satisfaction with the process of IVF treatment so far				
Very satisfied	n (%)	xx (xx.x)	xx (xx.x)	
Satisfied	n (%)	xx (xx.x)	xx (xx.x)	
Neither satisfied nor dissatisfied	n (%)	xx (xx.x)	xx (xx.x)	
Dissatisfied	n (%)	xx (xx.x)	xx (xx.x)	
Very dissatisfied	n (%)	xx (xx.x)	xx (xx.x)	
Missing	n	xx	xx	

Table 7: Adherence

		Frozen embryo transfer (n=XXX)	Fresh embryo transfer (n=XXX)
**Received frozen embryo transfer**	n (%)	xx (xx.x)	xx (xx.x)
Received frozen embryo transfer within 3 months of egg collection[10]	n (%)	xx (xx.x)	xx (xx.x)
Time from egg collection to frozen embryo transfer (days)	Mean {SD}	xx.x (x.xx)	xx.x (x.xx)
	Med [IQR]	xx [xx to xx]	xx [xx to xx]
	(Min to max)	xx (xx to xx)	xx (xx to xx)
Missing	n	xx	xx
**Received fresh embryo transfer**	n (%)	xx (xx.x)	xx (xx.x)

[10] Assuming 3 months = 92 days

Table 8: Unexpected serious adverse events by allocation

SAE number	Treatment allocation	Centre ID	Description	Severity	Related	Action taken	Outcome

Table 9: Protocol non-compliances

Protocol non-compliance number	Treatment allocation	Centre ID	Description	Reason
…	…	…	…	…
…	…	…	…	…
…	…	…	…	…
…	…	…	…	…
…	…	…	…	…
…	…	…	…	…

Secondary analyses

Table 10: Clinically important outcomes

		Frozen embryo transfer	Fresh embryo transfer	Effect estimate (99% CI)
**Total number of women with positive pregnancy test at 2 weeks ±3 days after embryo transfer**	N	xx	xx	
**Miscarriage**				RR (xx.x to xx.x)
Early (<12 weeks' gestation)	n (%)	xx (xx.x)	xx (xx.x)	
Late (12 to <24 weeks' gestation)	n (%)	xx (xx.x)	xx (xx.x)	
Missing	n	xx	xx	
**Multiple pregnancy**	n (%)	xx (xx.x)	xx (xx.x)	RR (xx.x to xx.x)
Missing	n	xx	xx	
**Total number of pregnant women with an ongoing pregnancy resulting in delivery**	N	xx	xx	
**Gestational diabetes mellitus**	n (%)	xx (xx.x)	xx (xx.x)	RR (xx.x to xx.x)
Missing	n	xx	xx	
**Multiple pregnancy**	n (%)	xx (xx.x)	xx (xx.x)	RR (xx.x to xx.x)
Missing	n	xx	xx	
**Hypertensive disorders**	n (%)	xx (xx.x)	xx (xx.x)	RR (xx.x to xx.x)
Chronic hypertension	n (%)	xx (xx.x)	xx (xx.x)	
Pregnancy induced hypertension	n (%)	xx (xx.x)	xx (xx.x)	
Pre-eclampsia	n (%)	xx (xx.x)	xx (xx.x)	
Eclampsia	n (%)	xx (xx.x)	xx (xx.x)	
Missing	n	xx	xx	
**Most severe hypertensive disorders**	n (%)	xx (xx.x)	xx (xx.x)	
Chronic hypertension	n (%)	xx (xx.x)	xx (xx.x)	
Pregnancy induced hypertension	n (%)	xx (xx.x)	xx (xx.x)	
Pre-eclampsia	n (%)	xx (xx.x)	xx (xx.x)	
Eclampsia	n (%)	xx (xx.x)	xx (xx.x)	
Missing	n	xx	xx	
**Antepartum haemorrhage**	n (%)			RR (xx.x to xx.x)
Placenta praevia	n (%)	xx (xx.x)	xx (xx.x)	
Placental abruption	n (%)	xx (xx.x)	xx (xx.x)	
Other	n (%)	xx (xx.x)	xx (xx.x)	
Missing	n	xx	xx	

		Frozen embryo transfer	Fresh embryo transfer	Effect estimate (99% CI)
**Preterm delivery (<37 completed weeks)**	n (%)	xx (xx.x)	xx (xx.x)	RR (xx.x to xx.x)
Missing	n	xx	xx	
**Very preterm delivery (<32 completed weeks)**	n (%)	xx (xx.x)	xx (xx.x)	RR (xx.x to xx.x)
Missing	n	xx	xx	
**Onset of labour**				
Spontaneous	n (%)	xx (xx.x)	xx (xx.x)	RR (xx.x to xx.x)
Induced	n (%)	xx (xx.x)	xx (xx.x)	
Planned caesarean section	n (%)	xx (xx.x)	xx (xx.x)	
Missing		xx	xx	
**Mode of delivery for each baby**	**N**	XX	XX	
Normal vaginal delivery	n (%)	xx (xx.x)	xx (xx.x)	RR (xx.x to xx.x)
Instrumental vaginal delivery	n (%)	xx (xx.x)	xx (xx.x)	RR (xx.x to xx.x)
Caesarean section	n (%)	xx (xx.x)	xx (xx.x)	RR (xx.x to xx.x)
Missing	n	xx	xx	
**Total number of babies born**[11]	N	XX	XX	
**Low birth weight (<2500 g at birth)**	n (%)	xx (xx.x)	xx (xx.x)	RR (xx.x to xx.x)
Missing	n	xx	xx	
**Very low birth weight (<1500 g at birth)**	n (%)	xx (xx.x)	xx (xx.x)	RR (xx.x to xx.x)
Missing	n	xx	xx	
**High birth weight (>4000 g at birth)**	n (%)	xx (xx.x)	xx (xx.x)	RR (xx.x to xx.x)
Missing	n	xx	xx	
**Large for gestational age (>90th centile)**	n (%)	xx (xx.x)	xx (xx.x)	RR (xx.x to xx.x)
Missing	n	xx	xx	
**Small for gestational age (<10th centile)**	n (%)	xx (xx.x)	xx (xx.x)	RR (xx.x to xx.x)
Missing	n	xx	xx	
**Congenital anomaly/birth defect**	n (%)	xx (xx.x)	xx (xx.x)	RR (xx.x to xx.x)
Missing	n	xx	xx	
**Perinatal mortality**	n (%)	xx (xx.x)	xx (xx.x)	RR (xx.x to xx.x)

[11] Analysis accounts for correlation between multiple births

		Frozen embryo transfer	Fresh embryo transfer	Effect estimate (99% CI)
Missing	n	xx	xx	

Table 11: Subgroup analyses for primary outcome

		Frozen embryo transfer	Fresh embryo transfer	Risk ratio (95% CI)	Interaction p-value
**Woman's age**					p
<35	n (%)	xx (xx.x)	xx (xx.x)	RR (xx.x to xx.x)	
35 to <40	n (%)	xx (xx.x)	xx (xx.x)	RR (xx.x to xx.x)	
≥40	n (%)	xx (xx.x)	xx (xx.x)	RR (xx.x to xx.x)	
**Fertility clinic**					p
1	n (%)	xx (xx.x)	xx (xx.x)	RR (xx.x to xx.x)	
2	n (%)	xx (xx.x)	xx (xx.x)	RR (xx.x to xx.x)	
3	n (%)	xx (xx.x)	xx (xx.x)	RR (xx.x to xx.x)	
4	n (%)	xx (xx.x)	xx (xx.x)	RR (xx.x to xx.x)	
5 …	n (%)	xx (xx.x)	xx (xx.x)	RR (xx.x to xx.x)	
**Stage of embryo at transfer**					p
Cleavage	n (%)	xx (xx.x)	xx (xx.x)	RR (xx.x to xx.x)	
Blastocyst	n (%)	xx (xx.x)	xx (xx.x)	RR (xx.x to xx.x)	
**Number of embryos transferred**					p
Single	n (%)	xx (xx.x)	xx (xx.x)	RR (xx.x to xx.x)	
Multiple	n (%)	xx (xx.x)	xx (xx.x)	RR (xx.x to xx.x)	
**Number of previous embryo transfers**					p
0	n (%)	xx (xx.x)	xx (xx.x)	RR (xx.x to xx.x)	
1–3	n (%)	xx (xx.x)	xx (xx.x)	RR (xx.x to xx.x)	
≥4	n (%)	xx (xx.x)	xx (xx.x)	RR (xx.x to xx.x)	
**Method of endometrial preparation for transfer**					N/A
Natural cycle	n (%)	xx (xx.x)	xx (xx.x)	N/A	
Hormone replacement cycle	n (%)	xx (xx.x)	xx (xx.x)	N/A	
**Method of embryo freezing**					N/A
Vitrification	n (%)	xx (xx.x)	xx (xx.x)	N/A	
Slow freezing	n (%)	xx (xx.x)	xx (xx.x)	N/A	

Table 12: Exploratory analysis

		Frozen embryo transfer (n=XXX)	Fresh embryo transfer (n=XXX)	Risk ratio (95% CI)
**Restricted analysis**				
Total number of couples, excluding those who did not receive the allocated intervention as randomised	N	xx	xx	
**Singleton baby born at term with appropriate weight for gestation**	n (%)	xx (xx.x)	xx (xx.x)	RR (xx.x to xx.x)
Missing	n	xx	xx	
**As-treated analysis**		xx	xx	
Total number of couples receiving each allocation	N			
**Singleton baby born at term with appropriate weight for gestation**	n (%)	xx (xx.x)	xx (xx.x)	RR (xx.x to xx.x)
Missing	n	xx	xx	

Document history

Version	Date	Edited by	Comments
0.1	12/15	MG	First draft version created December 2015.
0.2	22/12/15	AM	Review by Chief Investigator AM. Comments made.
0.3	01/16-02/16	MG	Updates made to dummy tables after CRF changes made.
0.4	02/16-03/16	MG	Updates made after meeting with CC, MG, PH and AM on 01/02/16.
0.5	03/16	MG	Updates made after meeting with CC, MG, PH and AM on 15/03/16.
0.6	03/16-05/16	MG	Updates made during/after meeting with CC, MG and AM on 31/03/16.
0.7	16/06/16	MG	Updates made after TC with SB, MG, PH and AM on 13/06/16.
0.8	24/06/16	MG	Updates made after meeting with SB, CC, MG, PH and AM on 21/06/16.
0.9	03/02/2017 – 28/03/2017	JB	Updated following changes to protocol (v1.7) and SAP.
0.10	27/04/2017 – 16/05/2017	JB	Updates following discussion with PH and AM on 10/04/17, and further guidance from AM and Louise Linsell (LL).
0.11	18/05/2017	JB	Updates following review by LL
0.12	19/05/2017	JB	Updated formatting to portrait
0.13	29/11/2019	JB	Updated following updates to statistical analysis plan
0.14	18/12/2019	JB	Removed sensitivity (CACE) analysis table (to be presented in text only)
0.15	19/05/2020	JB	Removed p-value column from primary outcome table, since this is not specified in SAP or protocol.
