# Supplementary material for: E-Freeze - a randomised controlled trial evaluating the clinical and cost effectiveness of a policy of freezing embryos followed by thawed frozen embryo transfer compared with a policy of fresh embryo transfer, in women undergoing in vitro fertilisation: a statistical analysis plan
Source: Trials. 2020 Jun 30;21:596. doi: 10.1186/s13063-020-04441-9 (PMC7329511; doi:10.1186/s13063-020-04441-9)
Supplement: Supplementary file 3 — Additional file 3. Appendix C – E-Freeze SAP v1.0.pdf. [file 13063_2020_4441_MOESM3_ESM.pdf]

E-Freeze
Funded by NIHR HTA Programme
ISRCTN: 61225414

Full title: Elective Freezing of embryos in assisted conception: a randomised controlled trial evaluating the clinical and cost-effectiveness of a policy of freezing embryos followed by thawed frozen embryo transfer, compared with a policy of fresh embryo transfer in women undergoing in vitro fertilisation

Statistical Analysis Plan

Version 1.0

19 July 2019

Authors: Melanie Greenland (E-Freeze Trial statistician 2015 to 2017, NPEU CTU)
Pollyanna Hardy (Senior statistician 2014 to 2017, NPEU CTU)
Jennifer Bell (E-Freeze Trial statistician 2017 to present, NPEU CTU)
Dr Louise Linsell (Senior statistician 2017 to present, NPEU CTU)

Reviewers: Dr Abha Maheshwari (Chief Investigator)
Ed Juszczak (Director NPEU CTU)
Professor Siladitya Bhattacharya (Director IAHS)

Protocol version: 2.0, 18 January 2017

Contents

1. Introduction 5
2. Background Information 6
2.1 Rationale 6
2.2 Objectives of the trial 6
2.3 Trial design 6
2.4 Eligibility 6
2.5 Interventions 7
2.6 Definition of primary and secondary outcomes 7
2.6.1 Primary outcome 7
2.6.2 Secondary outcomes 7
2.7 Hypothesis framework 8
2.8 Sample size and power 9
2.9 Intervention allocation 9
2.10 Data collection schedule 10
2.11 Interim analyses and stopping rules 11
2.12 Trial reporting 11
3. Protocol non-compliances 12
3.1 Major 12
3.2 Minor 12
4. Adherence to the intervention 13
5. Analysis populations 13
5.1 Post-randomisation exclusions 13
5.2 Population definitions 13
5.2.1 Intention to treat population 13
5.2.2 Interim analysis population 13
5.2.3 Safety population 13
6. Descriptive analyses 13
6.1 Representativeness of trial population and participant throughput 13
6.2 Baseline comparability of randomised groups 14
6.3 Loss to follow-up 15
6.4 Description of adherence to intervention 15
7. Comparative analysis 16
7.1 Detailed definition of outcomes 17
7.2 Primary analysis 23
7.3 Secondary analyses 23
7.4 Pre-specified subgroup analysis 24

7.5 Sensitivity analysis 24
7.6 Significance levels and adjustment of p-values for multiplicity 24
7.7 Missing data 25
7.8 Statistical software employed 25
8. Safety data analysis 25
9. Additional exploratory analysis 25
10. Deviation from analysis described in protocol 25
11. References 25
11.1 Trial documents 25
11.2 Other references 26
12. Approvals 27

List of abbreviations

AE	Adverse event
ANCOVA	Analysis of covariance
BMI	Body mass index
CACE	Complier-average causal effect
CI	Chief investigator
CONSORT	Consolidated standards of reporting trails
CRF	Case report form
CTU	Clinical trials unit
DMC	Data monitoring committee
eCRF	Electronic case report form
FSH	Follicle-stimulating hormone
g	Grams
GDM	Gestational diabetes mellitus
HFEA	Human Fertilisation and Embryology Authority
HTA	Health technology assessment
ICSI	Intracytoplasmic sperm injection
ITT	Intention to treat
IU	International units
IVF	In vitro fertilisation
kg	Kilogram
LMS	Lambda-Mu-Sigma
M	Metre
mm	Millimetre
NIHR	National Institute for Health Research
NNU	Neonatal unit
NPEU	National Perinatal Epidemiology Unit
OHSS	Ovarian hyperstimulation syndrome
RCOG	Royal College of Obstetricians and Gynaecologists
RCT	Randomised controlled trial
SAE	Serious adverse event
SAP	Statistical analysis plan
STAI	State-trait anxiety inventory
TSC	Trail steering committee
UK	United Kingdom

1. Introduction

This document details the proposed presentation and analyses for the main paper(s) reporting results from the National Institute for Health Research (NIHR) Health Technology Assessment (HTA) programme funded multicentre randomised controlled trial of fresh versus frozen embryo transfer for women undergoing in vitro fertilisation (E-Freeze).

The results reported in these papers will follow the strategy set out here. Subsequent analyses of a more exploratory nature will not be bound by this strategy, although they are expected to follow the broad principles described. The principles are not intended to curtail exploratory analysis (for example, to decide cut-points for categorisation of continuous variables), nor to prohibit accepted practices (for example, data transformation prior to analysis), but they are intended to establish the rules that will be followed, as closely as possible, when analysing and reporting the trial.

The analysis plan will be available on request when the principal papers are submitted for publication. Suggestions for subsequent analyses by journal editors or referees will be considered carefully and carried out, as far as possible, in line with the principles of this analysis plan.

Any deviations from the statistical analysis plan will be described and justified in the final report of the trial. The analysis should be carried out by an identified, appropriately qualified and experienced statistician, who should ensure the integrity of the data during their processing. Examples of such procedures include quality control and evaluation procedures.

2. Background Information

2.1 Rationale

In vitro fertilisation (IVF) involves several steps. Initially, hormones are used to stimulate a woman's ovaries to produce eggs which are harvested surgically. Next, embryos are created in the laboratory by mixing eggs with sperm produced by her partner, in conventional IVF; these are grown in culture for a few days before being replaced within the uterus by a process known as fresh embryo transfer. Spare embryos are usually frozen with a view to transfer at a later point in time – especially if the initial fresh transfer does not result in a pregnancy. Despite improvements in technology, IVF success rates remain low with an overall live birth rate of 25% per treatment. Additionally, there are concerns about health outcomes for mothers and babies conceived through IVF, particularly after fresh embryo transfer, including maternal ovarian hyperstimulation syndrome (OHSS) and perinatal morbidity.

It is believed that high levels of ovarian hormones during ovarian stimulation could create a relatively hostile environment for embryo implantation whilst increasing the risk of OHSS. It has been suggested that electively freezing embryos with the intention of thawing and replacing them within the uterus at a later stage (thawed frozen embryo transfer) instead of fresh embryo transfer, may lead to improved pregnancy rates and fewer complications. However, the existing evidence in support of an elective frozen embryo transfer policy, derived from three small randomised trials is inadequate to justify such a radical change in practice.

E-Freeze is a multi-centre randomised controlled trial (RCT) to determine if a policy of freezing embryos, followed by thawed frozen embryo transfer results in a higher healthy baby rate when compared with the current policy of transferring fresh embryos. The trial will recruit 1,086 couples undergoing their first, second or third cycle of IVF/ICSI/Split treatment at fertility centres in the UK.

2.2 Objectives of the trial

The primary objective of the trial is to determine if a policy of freezing embryos, followed by thawed frozen embryo transfer results in a higher healthy baby rate when compared with the current policy of transferring fresh embryos.

The secondary objectives of the trial are to assess if a policy of freezing embryos, followed by thawed frozen embryo transfer compared with the current policy of transferring fresh embryos results in:

1. Fewer complications associated with IVF/ICSI treatment and pregnancy
2. Greater cost-effectiveness from a health service and broader societal perspective

2.3 Trial design

Multi-centre, randomised controlled trial

2.4 Eligibility

Inclusion criteria:

- The female partner is ≥ 18 and < 42 years of age at the start of treatment (i.e. start of ovarian stimulation)
- Couples who are undergoing their 1st, 2nd or 3rd cycle of in vitro fertilisation (IVF) or intracytoplasmic sperm injection (ICSI) treatment
- Both partners are resident in the UK

- Both partners have provided written informed consent
- At least 3 good quality embryos on day 3 following fertilisation

Exclusion criteria:

- Donor gametes are used
- Pre-implantation genetic testing is planned
- Elective freezing of all embryos is planned for medical reasons (e.g. severe risk of OHSS/fertility preservation)
- Couples previously randomised to E-Freeze

2.5 Interventions

Standard care arm: Women will undergo fresh embryo transfer at the cleavage or blastocyst stage according to local protocols.

Intervention arm: All good quality embryos will be frozen according to local protocols. Women will be contacted by their embryologist/research nurse (or delegate) after randomisation and arrangements will be made for frozen embryo transfer (typically after 4 to 6 weeks and always within 3 months of the egg retrieval process). The couples will attend for a clinic visit and additional monitoring visits before thawed frozen embryo transfer is performed.

2.6 Definition of primary and secondary outcomes

2.6.1 Primary outcome

The primary outcome is a healthy baby.

A healthy baby is defined as a live singleton baby born at term (between 37 and 42 completed weeks of gestation) with an appropriate weight for gestation (weight between 10th and 90th centile for that gestation based on standardised charts).

2.6.2 Secondary outcomes

The secondary outcomes are separated by maternal safety, complications of pregnancy and delivery, measures of clinical effectiveness, measures of effectiveness of the process of freezing embryos and health economic outcome measures.

Maternal safety outcome

- Ovarian hyperstimulation syndrome (OHSS) - defined and classified as per the Royal College of Obstetricians and Gynaecologists (RCOG) green top guidelines[1].

Complications of pregnancy and delivery outcomes

- Vanishing twin or triplet (defined as either: more fetal heartbeats than babies born, more gestational sacs than babies born or more gestational sacs than fetal heartbeats)
- Miscarriage rate (defined as pregnancy loss prior to age of viability i.e. 24 weeks of gestation)
- Ectopic pregnancy
- Termination
- Gestational diabetes mellitus (GDM)
- Multiple pregnancy (defined as more than one fetal heartbeat or more than one gestational sac)

- Multiple births (including live and still births)
- Hypertensive disorders of pregnancy (chronic hypertension, pregnancy induced hypertension, pre-eclampsia and eclampsia)
- Most severe hypertensive disorder (from least to worst: chronic hypertension, pregnancy induced hypertension, pre-eclampsia and eclampsia)
- Antepartum haemorrhage (any bleeding per vaginum after 28 weeks of pregnancy including placenta praevia and placental abruption)
- Onset of labour (spontaneous, induced or planned caesarean section)
- Mode of delivery for each baby (normal vaginal delivery, instrumental vaginal delivery or caesarean section)
- Preterm delivery (defined as delivery at < 37 completed weeks)
- Very preterm delivery (defined as delivery at < 32 completed weeks)
- Low birth weight (defined as weight < 2500g at birth)
- Very low birth weight (defined as weight < 1500g at birth)
- High birth weight (defined as weight > 4000g at birth)
- Large for gestational age (defined as birth weight > 90th centile for gestational age at delivery, based on standardised charts)
- Small for gestational age (defined as birth weight < 10th centile for gestational age at delivery, based on standardised charts)
- Congenital anomaly/birth defect (all congenital anomalies/birth defects identified will be included)
- Perinatal mortality (stillbirth or late as well as early neonatal deaths, up to 28 days after birth)

Measures of clinical effectiveness outcomes

- Live birth rate (this is a live birth episode i.e. multiples will count as one)
- Singleton live birth rate
- Singleton live birth rate at term
- Singleton baby with appropriate weight for gestation
- Pregnancy rate (defined as positive pregnancy test at 2 weeks +/- 3 days after embryo transfer)
- Clinical pregnancy rate (defined as the presence of at least one fetal heartbeat at ultrasound between six and eight weeks gestation; ectopic pregnancy counts as a clinical pregnancy; multiple gestational sacs count as one clinical pregnancy)

Measures of the effectiveness of the process of freezing embryos outcomes

- Total number of embryos frozen, thawed and transferred for all randomised couples
- Proportion of thawed embryos that were then transferred for all randomised couples
- Failure of all embryos to survive after thawing leading to no embryo transfer

Health economic outcome measures

- Cost to the health service of treatment, pregnancy and delivery care
- Modelled long-term costs of health and social care, and broader societal costs

Other secondary outcomes

- Evaluation of emotional state (for both the female and male partners)

2.7 Hypothesis framework

This is a superiority trial and all comparisons will be analysed and presented on this basis.

2.8 Sample size and power

The proposed primary outcome for this trial is novel and is not currently reported by IVF clinics or national regulatory bodies. This means that a number of assumptions have been made in order to determine the expected event rate in the control arm (receiving current standard treatment), which may in turn result in a degree of imprecision in the estimate.

The most recent data from the HFEA[2], which collects data on all IVF cycles from all clinics in the UK, show that 25% of all women undergoing one episode of IVF treatment involving a fresh embryo transfer have a live birth, and 20% have singleton live births. These figures are for women of all age groups, not necessarily for women fulfilling the inclusion criteria for this trial in terms of the number of good quality embryos in their IVF cycle. No data are available regarding the healthy baby rate (live singletons born between 37 and 42 weeks' with appropriate weight for gestation), the primary outcome for this study. For our trial population we anticipate that the control arm event rate is likely to be less than 25%, possibly as low as 17%.

To provide relevant information regarding the event rate expected in the control arm, we surveyed 10 IVF centres that expressed an interest in the study, collecting data on the number of live births in women under the age of 42 undergoing their first IVF treatment in 2012. The average live birth episode rate from this survey was 31% with a 95% confidence interval of 25% to 37%. Accurate data on the healthy baby rate in those with at least 3 good quality embryos were not available. Although the live birth rate is expected to be higher in women with at least 3 good quality embryos (likely to have a better prognosis), we anticipate that the healthy baby rate in our trial population will be towards the lower end of the confidence interval, around 25%, taking into account the higher risk of preterm delivery and small for gestational age babies following IVF[3].

<u>The following assumptions have been made for the sample size calculation:</u>
We have assumed a healthy baby rate of between 17% and 25% in women eligible for the trial (age under 42 years with 3 good quality embryos) undergoing standard care (fresh embryo transfer). Taking into account the extra time, effort and potential expense involved in freezing embryos and the delay in embryo transfer of up to three months, a panel of clinicians across the UK agreed that the strategy of freezing embryos would be considered effective if the percentage of women having a healthy baby is increased by at least 8% in absolute terms. With 90% power and using a two-sided 5% level of statistical significance, we will need to randomise a total of 1,086 couples (543 in each group) in order to be able to detect an absolute difference of 8% from 17% to 25% and 9% from 25% to 34% in the healthy baby rate, between fresh embryo transfer and transfer of thawed frozen embryos. The difference detectable differs slightly depending on the event rate in the standard care group, which will be reviewed periodically by the Data Monitoring Committee (DMC).

It is a regulatory requirement for clinics in the UK to report live birth outcomes (including number, weight and gestation) after all embryo transfers i.e. there will be no loss to follow-up. Therefore we have not taken into account loss to follow-up for these sample size calculations.

It is anticipated that a proportion of those consented may not reach randomisation (not having 3 good quality day 3 embryos or requiring all embryos to be frozen for medical reasons), therefore a higher number will need to be consented. As there are no valid data to support the exact proportion, this will be monitored by DMC.

2.9 Intervention allocation

Randomisation will be performed after the creation of embryos, 3 days post egg collection. This will minimise the randomisation-to-intervention time interval as embryos are either transferred at the cleavage or blastocyst stage. Once all eligibility criteria are established (including ensuring that three or more good quality embryos are available), women will be randomised (allocation ratio 1:1) to a strategy of either:

Fresh embryo transfer

or

Elective freezing of embryos followed by thawing and replacement at a later date (typically 4 to 6 weeks later and always within 3 months of egg collection)

Randomisation will be undertaken by the research nurse or a delegated member of the research team using a secure web-based centralised system (with 24/7 telephone backup 365 days/year) hosted by the National Perinatal Epidemiology Unit Clinical Trials Unit (NPEU CTU), University of Oxford (ensuring allocation concealment). The randomisation will employ a minimisation algorithm to balance across the following factors: fertility clinic; woman's age (at the time of start of treatment i.e. ovarian stimulation): < 35 years, 35 to < 40 years, ≥ 40 years; primary/secondary infertility; self-reported duration of infertility: < 12 months, 12 to < 24 months, 24 to < 36 months, 36 to < 48 months, 48 to < 60 months, ≥ 60 months; method of insemination: IVF, ICSI, Split; and number of previous egg collections (cycles): 0, 1 and 2.

2.10 Data collection schedule

Data for both clinical and economic outcomes will be collected using bespoke electronic case report forms (eCRFs) and entered directly into the study's OpenClinica electronic database by the centre's research staff.

After consent and at embryo transfer, the couples will each complete a short paper-based questionnaire asking them how they are feeling. A short questionnaire will be provided for each partner to record details of time and travel expenses accrued during their treatment as part of the economic evaluation. This is to be completed at the time of embryo transfer.

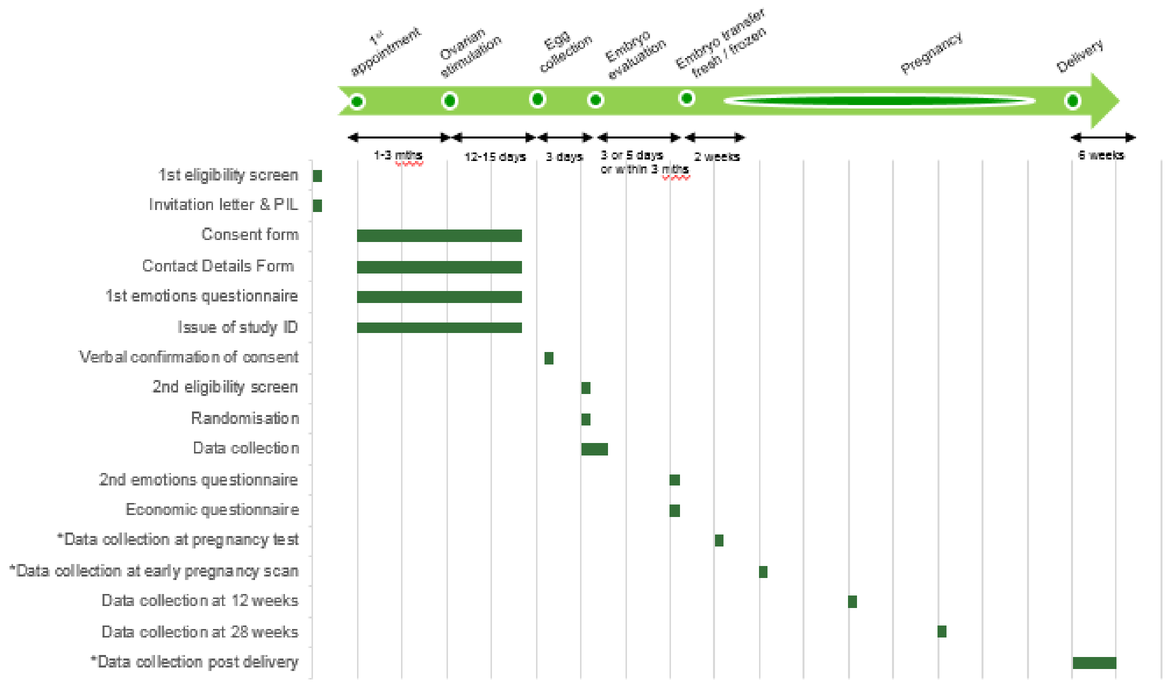

*Routinely collected as part of regulatory requirement

2.11 Interim analyses and stopping rules

An independent Data Monitoring Committee has been established, whose remit will be to safeguard the interests of trial participants, potential participants, their families, their carers, investigators, and the sponsor; to assess the safety and efficacy of the intervention during the trial, and monitor the trial's overall conduct, and protect its validity and credibility. The terms of reference for the DMC were agreed at their first meeting and the DMC Charter was finalised.

Interim analyses will be supplied, in strict confidence, to the DMC, as frequently as the Chair requests. The DMC will aim to meet in person at least annually, or more often as appropriate, and meetings should be timed so that reports can be fed into the Trial Steering Committee (TSC).

In the light of interim data and other evidence from relevant studies, the DMC will inform the TSC if, in its view, there is proof beyond reasonable doubt that the data indicate that the trial should be terminated. A decision to inform the TSC of such a finding will in part be based on statistical considerations. Appropriate proof beyond reasonable doubt cannot be specified precisely. A difference of at least 3 standard errors in the interim analysis of a major endpoint may be needed to justify halting or modifying the study prematurely. Unless modification or cessation of the trial is recommended by the DMC, the TSC, investigators, collaborators and administrative staff (except those who supply the confidential information) will remain ignorant of the results of the interim analysis. Collaborators and all others associated with the study may write to the DMC to draw attention to any concern they may have about the possibility of harm arising from the treatment under study.
The Trial Statistician will provide the DMC with interim reports unblinded to allocation.

2.12 Trial reporting

The trial will be reported according to the principles of the CONSORT statement.

3. Protocol non-compliances

A protocol non-compliance is defined as a failure to adhere to the protocol such as the wrong intervention being administered, incorrect data being collected and documented, errors in applying inclusion/exclusion criteria or missed follow-up visits due to error.

All protocol non-compliances will be listed in the final report. Non-compliances are defined below.

3.1 Major

The following are pre-defined major protocol non-compliances with a direct bearing on the primary outcome:

- Data considered to be fraudulent

3.2 Minor

The following will be defined as minor protocol non-compliances:

Participants randomised in error

These include couples:

- where the female partner is not ≥ 18 and < 42 years of age at the start of treatment (i.e. start of ovarian stimulation)
- who are not undergoing their 1st, 2nd or 3rd cycle of IVF or ICSI treatment
- who are not both resident in the UK
- for whom written informed consent is not provided from both partners
- who have less than three good quality embryos and/or the quality of embryos is assessed before or after day three following fertilisation
- for whom donor gametes are used
- for whom pre-implantation genetic testing is planned
- for whom elective freezing of all embryos is planned for medical reasons (e.g. severe risk of OHSS/fertility preservation)
- who have previously been randomised to E-Freeze

Participants who do not receive their intervention as allocated

These include couples:

- randomised to the freezing of embryos followed by thawing and replacement at a later date (within three months of the date of egg retrieval) but who do not receive frozen embryo transfer or receive it after three months of the date of egg retrieval (defined as three calendar months)
- who switch from their randomised trial arm to the other arm
- who do not receive any embryo transfer within three calendar months of the date of egg retrieval

Other minor protocol non-compliances

These include couples:

- randomised before or after day three after egg collection (where day zero is defined as the day of egg collection)
- for whom a pregnancy test was carried out outside two weeks +/- 3 days after embryo transfer
- for whom an ultrasound was performed before six weeks' or after eight weeks' gestation
- contacted by telephone outside 12 weeks' gestation +/- 14 days
- contacted by telephone outside 28 weeks' gestation +/- 14 days

4. Adherence to the intervention

Data will be collected on women allocated to frozen embryo transfer who received fresh embryo transfer or no embryos, and women allocated to fresh embryo transfer who received frozen embryo transfer or no embryos. Reasons for not receiving the allocated intervention will also be collected.

A sensitivity analysis will be conducted to compare the effects in compliers and non-compliers. See section 7.5.

The date of egg collection and embryo transfer will be collected to derive the time to embryo transfer, and non-adherence to the maximum of 3 months will be reported.

5. Analysis populations

5.1 Post-randomisation exclusions

Exclusions to the analysis post-randomisation are defined as any of the following:

- couples for whom written consent forms from both partners were not received
- couples for whom consent to use their data was withdrawn by one or both of the partners
- couples for whom an entire record of fraudulent data was detected (should fraudulent data be detected, consideration will be given to excluding all data for the site where such data were found)

The numbers of post-randomisation exclusions will be reported by randomised treatment group in a figure presenting the flow of participants, and reasons summarised.

5.2 Population definitions

5.2.1 Intention to treat population

The intention to treat (ITT) population will be all couples randomised, excluding post-randomisation exclusions.

5.2.2 Interim analysis population

Baseline characteristics will be presented for all couples with available data at the time of the database snapshot, excluding known post-randomisation exclusions. Secondary outcomes will be presented for all couples with available outcome data and women who have delivered at the time of database snapshot, excluding known post-randomisation exclusions.

5.2.3 Safety population

All couples randomised, excluding post-randomisation exclusions. Since the post-randomisation exclusions are for couples for whom no written consent has been obtained or who have withdrawn permission for us to use their data, the safety population must exclude these couples.

6. Descriptive analyses

6.1 Representativeness of trial population and participant throughput

The flow of participants through each stage of the trial will be summarised by randomised group using a figure presenting the flow of participants. This will describe the numbers of couples:

- Consented
- Not randomised (with reasons)
- Randomised
- Allocated to frozen embryo transfer (with reasons if did not receive allocation)
    - Received frozen embryo transfer
    - Did not receive frozen embryo transfer
        - Received fresh embryo transfer (with reasons)
        - No embryos transferred (with reasons)
- Allocated to fresh embryo transfer (with reasons if did not receive allocation)
    - Received fresh embryo transfer
    - Did not received fresh embryo transfer
        - Received frozen embryo transfer (with reasons)
        - No embryos transferred (with reasons)
- Randomised in error
- Withdrawn (consent/no consent to use data)
- Included in the ITT population
- Delivered (no. of women and no. of babies)
- Post-randomisation exclusions (with reasons)

6.2 Baseline comparability of randomised groups

Demographic and clinical characteristics at trial entry will be described for all couples in the ITT population by randomised group, and separately for couples who delivered (in the analysis report only). The following characteristics will be described:

- Fertility clinic
- Woman's age at ovarian stimulation (years)
- Woman's ethnicity
- Woman's smoking status
- Woman's BMI (kg/m^2)
- Type of infertility
- Woman's previous pregnancies
- Woman's previous live births
- Main cause of infertility
- Duration of infertility (months)
- Endoscratch performed
- Stimulation regimen used
- Total stimulation dose of FSH (IU)
- Adjuvants used
- Trigger injection used
- Total number of eggs collected
- Method of insemination
- Number of eggs fertilised normally (two pro nucleus)
- Time lapse used
- Good quality embryos created on day three
- Number of previous egg collections
- Number of previous embryo transfers

Clinical characteristics of the embryo and the endometrium, which are collected post-randomisation, will also be described for the ITT population by randomised group, and separately for couples who delivered (in the analysis report only):

- Method of embryo freezing
- Number of embryos frozen
- Time from egg collection to embryo freezing
- Number of embryos thawed
- Stage of embryo at transfer
- Number of embryos transferred
- Number of remaining frozen embryos after transfer
- Method of endometrial preparation for transfer
- Endometrial appearance
- Endometrial thickness (mm)

The number and percentage will be presented for binary and categorical variables. The mean and standard deviation or the median and the interquartile range will be presented for continuous variables, or the range if appropriate. There will be no tests of statistical significance performed nor confidence intervals calculated for differences between randomised groups on any baseline variable.

6.3 Loss to follow-up

Although there is no follow-up planned for this trial, the primary outcome is collected some time after trial entry. It can be expected that there may be some data missing due to the delay in data collection. The number and percentage of missing outcome data among couples will be reported for the two trial arms in the results tables. All maternal and infant deaths will be reported separately.

As it is a regulatory requirement for clinics in the UK to report live birth outcomes after all embryo transfers, there will be no loss to follow-up for the primary outcome. Therefore, a sensitivity analysis will not be conducted to impute missing data.

6.4 Description of adherence to intervention

Adherence to the allocated intervention will be presented for the ITT population. Adherence will be summarised by randomised group in a figure presenting the flow of participants.

The number of women allocated to frozen embryo transfer who received fresh embryo transfer or no embryos will be reported, along with the reason if provided. This will include the number of embryos failing to survive after thawing.

The number of women allocated to fresh embryo transfer who received frozen embryo transfer within three calendar months from the date of egg collection, or no embryos will be reported along with the reason if provided.

In addition, the number of women receiving frozen embryo transfer (whether allocated or not) within three months of egg collection will be presented, along with the mean and standard deviation or median and interquartile range of the time from egg collection to embryo transfer for those receiving frozen embryo transfer. The number of women receiving fresh embryo transfer (whether allocated or not) will also be presented.

7. Comparative analysis

Couples and babies will be analysed according to their allocation regardless of the intervention they actually received. The fresh embryo transfer group will be used as the reference group in all analyses.

Since we are following women up from prior to pregnancy until after pregnancy, this raises the question of the most appropriate denominator for outcomes that can only occur in particular states. There will be some outcomes that cannot be reported for some women (e.g. miscarriage for women who do not become pregnant, birthweight of babies for women who had a miscarriage). In order to perform the analyses for all outcomes on the ITT analysis population, these will not be considered as missing data and for these outcomes all women will be included in the denominator. This means that regardless of whether a pregnancy or live birth occurs, the woman will be included in the denominator once for all outcomes. Where this is a perinatal outcome, but there is no baby for the outcome to occur in, the woman will still be included once in the denominator.

Outcomes will be summarised with counts and percentages for categorical variables, means and standard deviations for normally distributed continuous variables, or median and interquartile range for other non-normally distributed continuous variables.

Risk ratios and confidence intervals will be calculated using log binomial regression, and if a model fails to converge a log Poisson regression model with a robust variance estimator will be used. Analyses will be adjusted for all minimisation factors where possible (i.e. fertility clinic, woman's age at the time of start of treatment (i.e. ovarian stimulation), primary/secondary infertility, self-reported duration of infertility, method of insemination and number of previous egg collections). Fertility clinic will be treated as a random effect in the model, and all other factors as fixed effects. Both crude and adjusted risk ratios will be presented, but the primary inference will be based on the adjusted estimates.

For neonatal secondary outcomes, the unit of analysis in the ITT analysis will be the mother. In cases of multiple pregnancy where the infants' outcomes differ, the worst outcome will be reported.

Analysis of secondary outcomes will be clearly delineated from the primary outcomes in any statistical reports produced.

The emotions questionnaires at randomisation and post-embryo transfer capture responses from the State-Trait Anxiety Inventory[5] (STAI). The response at randomisation will be used as a covariate in an analysis of covariance (ANCOVA) model. Hypothesis testing will investigate if there is a post-embryo transfer difference in the means of the two treatment groups having adjusted for responses at randomisation. To avoid bias, maximise the power of the study and to obey the intention-to-treat principle, the missing-indicator method[6] will be used to replace missing baseline scores. This method replaces all missing baseline observations with the same value and an extra indicator variable is added to the model to indicate whether the value for that variable is missing. Models will be fitted separately for both the female and male partner.

The following secondary outcomes will be described only and no formal statistical analysis comparing groups will be conducted:

- Chronic hypertension, pregnancy induced hypertension, pre-eclampsia, and eclampsia
- Most severe hypertensive disorder (from least to worst: chronic hypertension, pregnancy induced hypertension, pre-eclampsia, and eclampsia)

7.1 Detailed definition of outcomes

Outcome	Unit of analysis	Primary analysis denominator	Secondary analysis denominator (clinically relevant population)	Details of derivation
**Primary outcome**				
Healthy baby	Woman	ITT population		A healthy baby is defined as a live singleton baby born at term (between 37 and 42 completed weeks of gestation) with an appropriate weight for gestation (weight between 10th and 90th centile for that gestation based on standardised charts).<br><br>The LMS method[7] will be used to calculate birthweight centile.<br><br>Gestational age at birth is calculated as the difference between the expected date of delivery and 40 weeks, subtracted from the date of birth. This is divided by 7 to give the age in weeks.<br><br>Expected date of delivery is 38 weeks after egg collection for fresh transfer. For frozen transfer, the time between freezing and thawing is added on to this.
**Secondary outcomes**				
**Maternal safety outcome**				
Ovarian hyperstimulation syndrome (OHSS) - defined and classified as per the Royal College of Obstetricians and Gynaecologists (RCOG) green top guidelines	Woman	ITT population		
**Complications of pregnancy and delivery outcomes**				

Outcome	Unit of analysis	Primary analysis denominator	Secondary analysis denominator (clinically relevant population)	Details of derivation
Vanishing twin or triplet (defined as either: more fetal heartbeats than babies born, more gestational sacs than babies born, or more gestational sacs than fetal heartbeats)	Woman	ITT population	Total number of women with multiple gestational sacs and/or multiple fetal heartbeats	
Miscarriage rate (defined as pregnancy loss prior to age of viability i.e. 24 weeks of gestation)	Woman	ITT population	Total number of women with a positive pregnancy test at 2 weeks +/- 3 days after embryo transfer	Gestational age at a given date is calculated as the difference between the expected date of delivery and 40 weeks, subtracted from the given date. This is divided by 7 to give the age in weeks.<br><br>Expected date of delivery is 38 weeks after egg collection for fresh transfer. For frozen transfer, the time between freezing and thawing is added on to this.
Ectopic pregnancy	Woman	ITT population	Total number of women with a positive pregnancy test at 2 weeks +/- 3 days after embryo transfer	
Termination	Woman	ITT population	Total number of women with a positive pregnancy test at 2 weeks +/- 3 days after embryo transfer	
Gestational diabetes mellitus (GDM)	Woman	ITT population	Total number of pregnant women with an ongoing pregnancy resulting in delivery (live or stillbirth)	
Multiple pregnancy (defined as more than one fetal heartbeat or more than one gestational sac)	Woman	ITT population	Total number of women with a positive pregnancy test at 2 weeks +/- 3 days after embryo transfer	
Multiple births (including live and still births)	Woman	ITT population	Total number of pregnant women with an ongoing pregnancy resulting in delivery (live or stillbirth)	

Outcome	Unit of analysis	Primary analysis denominator	Secondary analysis denominator (clinically relevant population)	Details of derivation
Hypertensive disorders of pregnancy (chronic hypertension, pregnancy induced hypertension, pre-eclampsia, and eclampsia)	Woman	ITT population	Total number of pregnant women with an ongoing pregnancy resulting in delivery (live or stillbirth)	
Most severe hypertensive disorder (from least to worst: chronic hypertension, pregnancy induced hypertension, pre-eclampsia, and eclampsia)	Woman	ITT population	Total number of pregnant women with an ongoing pregnancy resulting in delivery (live or stillbirth)	
Antepartum haemorrhage (any bleeding per vaginum after 28 weeks of pregnancy including placenta praevia and placental abruption)	Woman	ITT population	Total number of pregnant women with an ongoing pregnancy resulting in delivery (live or stillbirth)	
Onset of labour (spontaneous, induced or planned caesarean section)	Woman	ITT population	Total number of pregnant women with an ongoing pregnancy resulting in delivery (live or stillbirth)	
Mode of delivery for each baby (normal vaginal delivery, instrumental vaginal delivery or caesarean section)	Woman	ITT population	Total number of infants of pregnant women with an ongoing pregnancy resulting in delivery (live or stillbirth)	
Preterm delivery (defined as delivery at < 37 completed weeks)	Woman	ITT population	Total number of pregnant women with an ongoing pregnancy resulting in delivery (live or stillbirth)	Gestational age at birth is calculated as the difference between the expected date of delivery and 40 weeks, subtracted from the date of birth. This is divided by 7 to give the age in weeks.<br><br>Expected date of delivery is 38 weeks after egg collection for fresh transfer. For frozen transfer, the time between freezing and thawing is added on to this.
Very preterm delivery (defined as delivery at < 32 completed weeks)	Woman	ITT population	Total number of pregnant women with an ongoing pregnancy resulting in delivery (live or stillbirth)	Gestational age at birth is calculated as the difference between the expected date of delivery and 40 weeks, subtracted from the date of birth. This is divided by 7 to give the age in weeks.

Outcome	Unit of analysis	Primary analysis denominator	Secondary analysis denominator (clinically relevant population)	Details of derivation
				Expected date of delivery is 38 weeks after egg collection for fresh transfer. For frozen transfer, the time between freezing and thawing is added on to this.
Low birth weight (defined as weight < 2500g at birth)	Infant	ITT population	Total number of babies born	
Very low birth weight (defined as weight < 1500g at birth)	Infant	ITT population	Total number of babies born	
High birth weight (defined as weight > 4000g at birth)	Infant	ITT population	Total number of babies born	
Large for gestational age (defined as birth weight > 90th centile for gestational age at delivery, based on standardised charts)	Infant	ITT population	Total number of babies born	Using LMS method for birthweight centiles.<br><br>Gestational age at birth is calculated as the difference between the expected date of delivery and 40 weeks, subtracted from the date of birth. This is divided by 7 to give the age in weeks.<br><br>Expected date of delivery is 38 weeks after egg collection for fresh transfer. For frozen transfer, the time between freezing and thawing is added on to this.
Small for gestational age (defined as birth weight < 10th centile for gestational age at delivery, based on standardised charts)	Infant	ITT population	Total number of babies born	Using LMS method for birthweight centiles.<br><br>Gestational age at birth is calculated as the difference between the expected date of delivery and 40 weeks, subtracted from the date of birth. This is divided by 7 to give the age in weeks.<br><br>Expected date of delivery is 38 weeks after egg collection for fresh transfer. For frozen transfer, the time between freezing and thawing is added on to this.
Congenital anomaly/birth defect (all congenital anomalies/birth defects identified will be included)	Infant	ITT population	Total number of babies born	

Outcome	Unit of analysis	Primary analysis denominator	Secondary analysis denominator (clinically relevant population)	Details of derivation
Perinatal mortality (stillbirth or late as well as early neonatal deaths, up to 28 days after birth)	Infant	ITT population	Total number of babies born	
**Measures of clinical effectiveness outcomes**				
Live birth rate (this is a live birth episode i.e. twins will count as one)	Woman	ITT population		
Singleton live birth rate	Woman	ITT population		
Singleton live birth rate at term	Woman	ITT population		Gestational age at birth is calculated as the difference between the expected date of delivery and 40 weeks, subtracted from the date of birth. This is divided by 7 to give the age in weeks.<br><br>Expected date of delivery is 38 weeks after egg collection for fresh transfer. For frozen transfer, the time between freezing and thawing is added on to this.
Singleton baby with appropriate weight for gestation	Woman	ITT population		The LMS method will be used to calculate birthweight centiles, which will be between the 10th and 90th centile.<br><br>Gestational age at birth is calculated as the difference between the expected date of delivery and 40 weeks, subtracted from the date of birth. This is divided by 7 to give the age in weeks.<br><br>Expected date of delivery is 38 weeks after egg collection for fresh transfer. For frozen transfer, the time between freezing and thawing is added on to this.
Pregnancy rate (defined as positive pregnancy test at 2 weeks +/- 3 days after embryo transfer)	Woman	ITT population		

Outcome	Unit of analysis	Primary analysis denominator	Secondary analysis denominator (clinically relevant population)	Details of derivation
Clinical pregnancy rate (defined as the presence of at least one fetal heartbeat at ultrasound between six and eight weeks gestation; ectopic pregnancy counts as a clinical pregnancy; multiple gestational sacs count as one clinical pregnancy)	Woman	ITT population		
**Measures of the effectiveness of the process of freezing embryos outcomes**				
Total number of embryos frozen, thawed and transferred for all randomised couples	Couple	Total number of randomised couples		
Proportion of thawed embryos that were then transferred for all randomised couples	Couple	Total number of randomised couples		
Failure of all embryos to survive after thawing leading to no embryo transfer	Couple	ITT population		
**Other secondary outcomes**				
Evaluation of emotional state (for both the female and male partners)	Man<br>Woman	Total number of randomised couples		

7.2 Primary analysis

The primary analysis for the primary outcome and all secondary outcomes will be conducted on the ITT population.

7.3 Secondary analyses

There are a number of other clinically relevant populations of equal importance to the ITT population in the interpretation of results. In order to assess the treatment effect for the clinically relevant populations (e.g. effect on live birth rate for women who become pregnant), secondary analyses of specific outcomes will be performed with respect to clinically relevant denominators. These are as follows:

Per total number of women with a positive pregnancy test at 2 weeks +/- 3 days after embryo transfer:

- Miscarriage rate (defined as pregnancy loss prior to age of viability i.e. 24 weeks of gestation)
- Multiple pregnancy (defined as more than one fetal heartbeat or more than one gestational sac)

Per total number of pregnant women with an ongoing pregnancy resulting in delivery:

- Gestational diabetes mellitus
- Multiple births (including live and still births)
- Hypertensive disorders of pregnancy (chronic hypertension, pregnancy induced hypertension, pre-eclampsia and eclampsia)
- Most severe hypertensive disorders (from least to worst: chronic hypertension, pregnancy induced hypertension, pre-eclampsia and eclampsia)
- Antepartum haemorrhage (any bleeding per vaginum after 28 weeks of pregnancy including placenta praevia and placental abruption)
- Preterm delivery (defined as delivery at < 37 completed weeks)
- Very preterm delivery (defined as delivery at < 32 completed weeks)
- Onset of labour (spontaneous, induced or planned caesarean section)
- Mode of delivery for each baby (normal vaginal delivery, instrumental vaginal delivery or caesarean section)

Per total number of babies born:

- Low birth weight (defined as weight < 2500g at birth)
- Very low birth weight (defined as weight < 1500g at birth)
- High birth weight (defined as weight > 4000g at birth)
- Large for gestational age (defined as birth weight > 90th centile for gestational age at delivery, based on standardised charts)
- Small for gestational age (defined as birth weight < 10th centile for gestational age at delivery, based on standardised charts)
- Congenital anomaly/birth defect (all congenital anomalies/birth defects identified will be included)
- Perinatal mortality (stillbirth or late as well as early neonatal deaths, up to 28 days after birth).

For neonatal secondary outcomes, where the unit of analysis is the baby, the method of analysis will account for the anticipated correlation in outcomes between multiples.

7.4 Pre-specified subgroup analysis

The consistency of the effect of type of embryo transfer across specific subgroups of couples will be assessed for the primary outcome using the statistical test of interaction, in addition to the adjusted model. Results will be presented on forest plots with risk ratios, 95% confidence intervals and the results of the interaction test.

Pre-specified subgroup analyses are:

- Fertility clinic
- Woman's age (at the time of start of treatment i.e. ovarian stimulation): < 35 years, 35 to <40 years, ≥ 40 years
- Blastocyst versus cleavage
- Single versus multiple embryo transfer
- Number of previous embryo transfers (0, 1 – 3, ≥ 4).

In addition, for those receiving frozen embryo transfer, the following subgroups will be described using numbers and percentages:

- Natural versus hormone replacement cycles
- Vitrification versus slow freezing.

7.5 Sensitivity analysis

To assess the impact of non-compliance to the randomised allocation, i.e. women randomised to the frozen arm receiving fresh embryo transfer (non-compliers), a complier-average causal effect (CACE) analysis will be conducted. This analytic technique provides a robust estimate of the treatment effect amongst compliant participants.[8, 9]

The baseline characteristics of women randomised to the frozen arm will be reported by compliance status, and the unadjusted event rate for the primary outcome will be calculated for the observed compliers and non-compliers in the frozen arm. CACE analysis assumes that the proportion of would-be non-compliers in the fresh arm (i.e. couples in the fresh group who would not have complied had they been randomised to frozen) is the same as the proportion of non-compliers in the frozen group. It also assumes that the event rate among the non-compliers in the frozen arm is the same as the event rate among the would-be non-compliers in the fresh group. Applying these two assumptions, the unadjusted event rate for the primary outcome will be calculated for the would-be compliers and would-be non-compliers in the fresh arm. The unadjusted CACE risk ratio plus 95% confidence intervals for the primary outcome will be calculated using the event rates for compliant groups only (i.e. the observed compliers in the frozen arm and the would-be compliers in the fresh arm). The confidence intervals for the CACE estimated risk ratio will be calculated using bootstrapping methods.[10]

If an entire record is considered fraudulent, this will be excluded from all analyses (see section 5.1). However, if any individual data is considered fraudulent, a sensitivity analysis will be conducted on outcomes where any fraudulent data is detected excluding data considered fraudulent.

7.6 Significance levels and adjustment of p-values for multiplicity

For all analyses of the primary outcome, a 95% confidence interval will be calculated. To take account of the number of hypothesis tests performed, 99% confidence intervals will be used for all analyses of the secondary outcomes.

7.7 Missing data

Missing data will be described, for example, by presenting the number of individuals in the missing category. All data collected on data collection forms will be used, since only essential data items will be collected.

For any partially completed self-evaluated STAI questionnaires in the Emotions Questionnaire CRF, the following strategy for estimation of total and subscale scores will be employed where items are missing:

> *If one or two items are omitted then the prorated full-scale score can be obtained by: Determining the mean weighted score for the scale items to which the individual responded, multiply this value by 20 and then round the product to the next higher whole number. If three or more items are omitted then the validity of the questionnaire must be questioned.*[5]

7.8 Statistical software employed

The statistical software Stata/SE will be used for all analyses.

8. Safety data analysis

Serious adverse events will be listed by allocation.

9. Additional exploratory analysis

The following exploratory analyses will be conducted on the primary outcome:

- A restricted per protocol analysis, excluding couples who did not receive the allocated intervention as randomised.
- An as-treated analysis, grouping couples according to the allocation they received.

Analysis methods as used for the primary analysis will be employed.

Any other additional exploratory analyses not specified in the analysis protocol will be exploratory in nature and a 1% two-sided significance level will be used with 99% confidence intervals. All such analyses will be approved by the Co-investigator Group.

10. Deviation from analysis described in protocol

None yet.

11. References

11.1 Trial documents

Dummy tables can be found in K:\NPEUDATA\Current trials\E-Freeze\Statistics\Dummy Tables\Final Analysis

The Data Derivation spreadsheet can be found in K:\NPEUDATA\Current trials\E-Freeze\Statistics\Data derivation

11.2 Other references

1. Royal College of Obstetrics and Gynaecology. Ovarian Hyperstimulation Syndrome, Management (Green-Top Guideline No. 5). Accessed: [24/08/15]. Available from: https://www.rcog.org.uk/en/guidelines-research-services/guidelines/gtg5/.
2. Human Fertilization and Embryology Authority. Fertility Treatment 2012 - Trends & Figures. Accessed: [24/08/15]. Available from: http://www.hfea.gov.uk/docs/FertilityTreatment2012TrendsFigures.PDF.
3. Pandey S, Shetty A, Hamilton M, Bhattacharya S, Maheshwari A. Obstetric and Perinatal Outcomes in Singleton Pregnancies Resulting from IVF/ICSI: A Systematic Review and Meta-Analysis. Hum Reprod Update. 2012;18(5):485–503.
4. Yelland LN, Salter AB, Ryan P. Relative Risk Estimation in Randomized Controlled Trials: A Comparison of Methods for Independent Observations. Int J Biostat 2011 (7). DOI: 10.2202/1557–4679.1278.
5. Spielberger, C. D., Gorsuch, R. L., Lushene, R., Vagg, P. R., & Jacobs, G. A. (1983). *Manual for the State-Trait Anxiety Inventory*. Palo Alto, CA: Consulting Psychologists Press.
6. White IR, Thompson SG. Adjusting for partially missing baseline measurements in randomized trials. Stat Med 2005; 24:993-1007.
7. Cole TJ, Freeman JV, Preece MA. British 1990 growth reference centiles for weight, height, body mass index and head circumference fitted by maximum penalized likelihood. Stat Med 1998; 17(4): 407-29.
8. Dunn G, Maracy M, Dowrick C, Ayuso-Mateos JL, Dalgard OS, Page H et al (2003). Estimating psychological treatment effects from a randomised controlled trial with both non-compliance and loss to follow-up. British Journal of Psychiatry 2003; 183: 323-331.
9. Hewitt CE, Torgerson DJ, Miles JNV. Is there another way to take account of noncompliance in randomized controlled trials? Canadian Medical Association Journal 2006; 175(4):347-348.
10. Opondo C, Halliday K, Witek-McManus S, Allen E. Estimating intervention effect in cluster randomised controlled trials with non-compliance. Trials 2017; 18.

12. Approvals

Senior Statistician	Name: Dr Louise Linsell	
	Signature	Date 31/7/19
Chief Investigator	Name: Dr Abha Maheshwari	
	Signature	Date 1/8/19
Chair of Trial Steering Committee (or delegate)	Name: Professor Richard Anderson	
	Signature	Date 25/7/19

Document history

Version	Date	Edited by	Comments/justification	Timing in relation to interim analysis / unblinding
0.1	April/May 16	MG	First draft version created April/May 2016.	Pre unblinding
0.2	May 16	MG	Updates made after review by PH on 25 May 2016.	Pre unblinding
0.3	June 16	PH	Further updates in preparation for TC with CI.	Pre unblinding
0.4	16 June 16	MG	Updates made after TC with SB, MG, PH and AM on 13 June 2016.	Pre unblinding
0.5	24 June 16	MG	Updates made after meeting with SB, CC, MG, PH and AM on 21 June 2016.	Pre unblinding
0.6	21 Dec 16	MG	Updates made after SAP meeting with CC, MG, PH, EJ and AM on 4 Oct 2016 and after protocol updates to include 2nd and 3rd cycles.	Pre unblinding
0.7	25 Jan 17	JB	Minor comments and edits	Pre unblinding
0.8	20 Mar 2017	PH	Updated to be in line with new SAP SOP and template	Pre unblinding
0.9	21-Mar – 7 Apr 17	PH/JB	Updates after discussions between PH and JB on 21/3/17. Now includes table of outcomes specifying analysis units, denominators and derivations for each outcome.	Pre unblinding
0.10	24 Apr 2017	JB	Updated section 7.1, with mind amendments and clarifications to	Pre unblinding

			other sections, following discussion with AM on 10 April 2017	
0.11	16-18 May 2017	JB	Amendments following review by Louise Linsell.	Pre unblinding
0.12	25 July 2017	JB	Minor amendments following review by Data Monitoring Committee on 13 July 2017.	Unblinded on 15 June 2017
0.13	12 September 2017	LL, JB	Sensitivity Analysis & Additional Exploratory Analysis sections updated.<br>Comments removed	Unblinded on 15 June 2017
0.14	17 June 2019	LL, JB	Minor changes following comments from TSC.<br>Clarification to section 7.5 Sensitivity Analysis, and addition of references.	Unblinded on 15 June 2017
1.0	19 July 2019	JB	Final version for approval.	Unblinded on 15 June 2017
